# The anaphase promoting complex impacts repair choice by protecting ubiquitin signalling at DNA damage sites

**DOI:** 10.1038/ncomms15751

**Published:** 2017-06-12

**Authors:** Kyungsoo Ha, Chengxian Ma, Han Lin, Lichun Tang, Zhusheng Lian, Fang Zhao, Ju-Mei Li, Bei Zhen, Huadong Pei, Suxia Han, Marcos Malumbres, Jianping Jin, Huan Chen, Yongxiang Zhao, Qing Zhu, Pumin Zhang

**Affiliations:** 1 National Center for Protein Sciences Beijing, Life Sciences Park, Beijing 102206, China; 2 State Key Laboratory of Proteomics, Beijing Proteome Research Center, Beijing Institute of Radiation Medicine, 27 Taiping Road, Beijing 100850, China; 3 Department of Molecular Physiology and Biophysics, Baylor College of Medicine, Houston, Texas 77030, USA; 4 Department of Medical Oncology, The Second Affiliated Hospital of Xi'an Jiaotong University Medical College, Xi'an, Shaanxi 710004, China; 5 Department of Radiation Oncology, The First Affiliated Hospital of Xi'an Jiaotong University Medical College, Xi'an, Shaanxi 710061, China; 6 National Center for International Research of Biological Targeting Diagnosis and Therapy, and Collaborative Innovation Center for Targeting Tumor Diagnosis and Therapy, Guangxi Medical University, Guangxi 530021, China; 7 Department of Biochemistry and Molecular Biology, University of Texas Health Sciences Center, Houston, Texas 77030, USA; 8 Spanish National Cancer Research Centre (CNIO), Melchor Fernández Almagro 3, Madrid, Spain

## Abstract

Double-strand breaks (DSBs) are repaired through two major pathways, homology-directed recombination (HDR) and non-homologous end joining (NHEJ). While HDR can only occur in S/G2, NHEJ can happen in all cell cycle phases (except mitosis). How then is the repair choice made in S/G2 cells? Here we provide evidence demonstrating that APC^Cdh1^ plays a critical role in choosing the repair pathways in S/G2 cells. Our results suggest that the default for all DSBs is to recruit 53BP1 and RIF1. BRCA1 is blocked from being recruited to broken ends because its recruitment signal, K63-linked poly-ubiquitin chains on histones, is actively destroyed by the deubiquitinating enzyme USP1. We show that the removal of USP1 depends on APC^Cdh1^ and requires Chk1 activation known to be catalysed by ssDNA-RPA-ATR signalling at the ends designated for HDR, linking the status of end processing to RIF1 or BRCA1 recruitment.

## Abstract (web summary)

The choice between homologous recombination and non-homologous end-joining is largely influenced by cell cycle. Here the authors show that APCCdh1 promotes homologous recombination by removing USP1, allowing polyubiquitinated histones to recruit BRCA1.

Genome stability is constantly challenged by DNA damage resulted from DNA replication errors and attacks by internal and external agents. Among many forms of DNA damage, double-strand breaks (DSBs) are the most dangerous to the integrity of the genome. DSBs are repaired through two pathways, homology-directed recombination (HDR) and non-homologous end joining (NHEJ)^1^,^2^,^3^. The choice between these two pathways is largely influenced by cell cycle phases, with NHEJ primarily occurring in G1 and HDR in S/G2 when homologous sequences are available from sister chromosomes^3^,^4^.

The anaphase-promoting complex/cyclosome (APC/C) is an E3 ubiquitin ligase critical for mitotic progression^5^. It utilizes two adaptor proteins, Cdc20 and Cdh1 (Fzr1), to bring in substrates for ubiquitination (K11-linked)^6^. Cdc20 functions primarily in mitosis whereas Cdh1 functions in other phases of the cell cycle, especially in G1 to prevent precocious S phase entry^7^. Besides the function in cell cycle regulation, APC^Cdh1^ has been implicated in DNA damage response. It was reported to mediate ubiquitination and degradation of USP1 to allow NER repair of UV-induced DNA damage^8^, Rad17 for checkpoint termination at the end of UV-induced DNA damage response^9^ and Plk1 to prevent precocious mitotic entry^10^. More recently, APC^Cdh1^ was proposed to regulate the clearance of CtIP, an essential HR protein, at a late time of HDR repair to prevent excessive end resection for optimal HR efficiency^11^.

The binding of Cdh1 to APC is regulated by phosphorylation of Cdh1. From mitotic exit to sometime before the restriction point in G1, Cdh1 remains in a hypo-phosphorylated (thus active form)^12^,^13^. During this time window, its substrates including USP1 and CtIP are degraded. However, it is phosphorylated by CDKs afterwards and becomes inactive. In budding yeast, it is the Cdc14 phosphatase that dephosphorylates (and activates) Cdh1 among many proteins phosphorylated by CDK1 upon mitotic entry^14^,^15^. There are two mammalian homologues of Cdc14, namely Cdc14A and B. Like Cdc14, Cdc14B is a nucleolar protein, but Cdc14A’s localization remains elusive, although it was initially reported to localize to and regulate the function of centrosomes^16^,^17^. In contrast to budding yeast, neither Cdc14A nor B is required for mitotic exit^18^,^19^. Instead, it is another phosphatase, PP2A-B55α, that promotes mitotic exit in mammalian cells^20^. It remains unclear which phosphatase dephosphorylates Cdh1 during mitotic exit or in other phases of the cell cycle. However, accumulating evidence suggest that in response to DNA damage it is Cdc14B that activates Cdh1 (refs 10, 21). DNA damage induces Cdc14B translocation from nucleolus to the nucleoplasm^10^, and it has been shown that the translocation is Chk1-dependent^22^. More recently, we showed that Cdc14A and B functioned redundantly in both HR- and NHEJ-mediated DNA damage repair, likely through dephosphorylating Cdh1 (ref. 21).

Dynamic ubiquitination and de-ubiquitination is known to be important in transmitting DNA damage signals and in regulating various steps in repair^23^. Upon DNA DSB, ATM is activated and initiates a series of phosphorylation events that ultimately result in the recruitment of two E3 ubiquitin liagases, RNF8 first and then RNF168 (ref. 24). RNF168 catalyses the formation of K63-linked poly-Ub chains on H2A/H2B which then signal BRCA1 (mainly its A-complex) recruitment^25^; and RNF168 also contributes to the recruitment of 53BP1 through helping the exposure of H4K20me2 (refs 26, 27). BRCA1 promotes homologous recombination by further recruiting BRCA2, RAD51, and so on (refs 28, 29), while 53BP1 promotes NHEJ by recruiting RIF1 (refs 30, 31). RIF1 pushes for NHEJ repair by preventing BRCA1 recruitment via unknown mechanisms^30^,^32^. On the other hand, BRCA1 can also prevent RIF1 recruitment through CtIP^31^ and/or UHRF1 (ref. 33). Thus, RIF1 and BRCA1 expel each other from damage sites and which protein prevails is influenced by the cell cycle stage^31^. In G1, NHEJ dominates probably because CtIP is at a low level and is not phosphorylated, resulting in BRCA1 unable to expel RIF1, but from S phase onwards, BRCA1 gains the ability to expel RIF1 and promote HR as CtIP and UHRF1 becomes phosphorylated by CDK^31^,^33^. However, despite this antagonizing relationship between BRCA1 and RIF1, RIF1 IRIF (irradiation-induced foci) and BRCA1 IRIF co-exist in S/G2 cells, and in fact, S/G2 cells can also perform NHEJ repair^34^,^35^,^36^,^37^. What then decides which DSB gets repaired through HDR and which through NHEJ in an S/G2 cell? Here we provide evidence demonstrating that APC^Cdh1^ plays a critical role in choosing the repair pathways in S/G2. Our results suggest that the default for all DSBs in S/G2 is to recruit 53BP1 and RIF1, but not BRCA1. BRCA1 is blocked because its recruiting signal, K63-linked poly-ubiquitin chains on histones, is actively destroyed by the deubiquitinating enzyme USP1. USP1 is recruited to damage sites in an MDC1-dependent manner. We show that the removal of USP1 depends on APC^Cdh1^ and the activation of this E3 ubiquitin ligase is linked to end processing through Chk1 activation.

## Results

### RIF1 and BRCA1 IRIF co-exist in S/G2 cells

**Figure 1:**
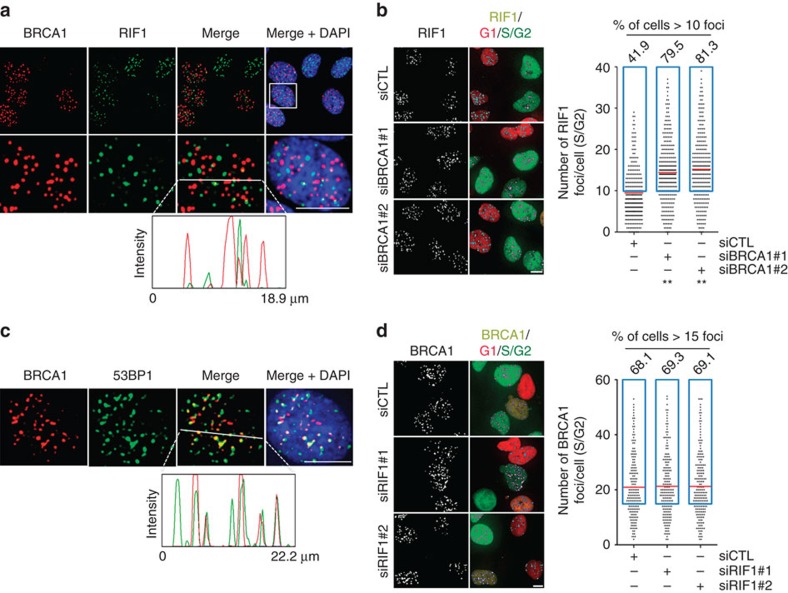
RIF1 and BRCA1 IRIF co-exist in S/G2 cells. (**a**) Co-staining of BRCA1 and RIF1 in U2OS cells 1 h after 2 Gy IR. (**b**) Quantification of RIF1 focus number per cell in U2OS-Fucci cells transfected with control or two different BRCA1 siRNAs. The cells were subjected to 2 Gy IR 3 days after the siRNA transfection. Left panel: representative images of RIF1 foci. Right panel: quantification of RIF1 focus number in the cells (*n*>212). (**c**) Co-staining of BRCA1 and 53BP1 in U2OS cells 1 h after 2 Gy IR. (**d**) Quantification of BRCA1 focus per cell in U2OS-Fucci cells transfected with control or two different RIF1 siRNAs. Cells were subjected to 2 Gy IR 3 days after the siRNA transfection. Left panel: representative images of BRCA1 foci. Right panel: quantification of BRCA1 focus number in the cells (*n*>188). The intensity profile graphs show the immunofluorescence intensities of the indicated proteins along the line on the images (**a**,**c**). Red lines on the graphs indicate the mean number of RIF1 (**b**) or BRCA1 (**d**) foci per cell. Blue boxes designate cells with more than 10 RIF1 foci (**b**) or 15 BRCA1 foci (**d**). The percentages of RIF1-positive (>10 foci) or BRCA1-positive (>15 foci) cells are indicated above each blue box. ** (in **b**) indicates *P*<0.001 (student’s *t*-test). Scale bars represent 10 μm.

In S/G2 cells, both HDR and NHEJ can occur^34^,^35^. Indeed, RIF1 (a marker for NHEJ) and BRCA1 (a marker for HDR) foci co-exist but do not co-localize in S/G2 cells (Fig. 1a), consistent with the notion that BRCA1 and RIF1 antagonize each other^30^,^31^. We refer RIF1 foci as Pro-NHEJ and BRCA1 foci as Pro-HDR thereafter. As expected, all RIF1 IRIF colocalize with that of DNA-PKcs (Supplementary Fig. 1a). It was reported previously^30^,^31^ that depletion of BRCA1 results in an increase in the number of RIF1 foci in S/G2 cells (Fig. 1b and Supplementary Fig. 1b). In other words, if a DSB were not occupied by BRCA1, RIF1 would take the place, suggesting that there is nothing at a Pro-HDR DSB, except BRCA1, that can prevent RIF1 from being recruited. Consistent with that, 53BP1, a factor required for RIF1 recruitment^30^,^31^, co-localizes with BRCA1 as well as RAD51 in some but not all IRIF (irradiation induced foci) (Fig. 1c and Supplementary Fig. 1c). On the other hand, the reverse is not true. The depletion of RIF1 did not affect BRCA1 focus number in S/G2 cells (Fig. 1d), suggesting that Pro-NHEJ DSBs are not competent to recruit BRCA1. However, as reported previously^31^, depletion of RIF1 did allow BRCA1 focus formation in G1 (Fig. 1d), indicating that the Pro-NHEJ sites in G1 are different than those in S/G2.

### RIF1 IRIF in S/G2 cells lack poly-Ub modification

**Figure 2:**
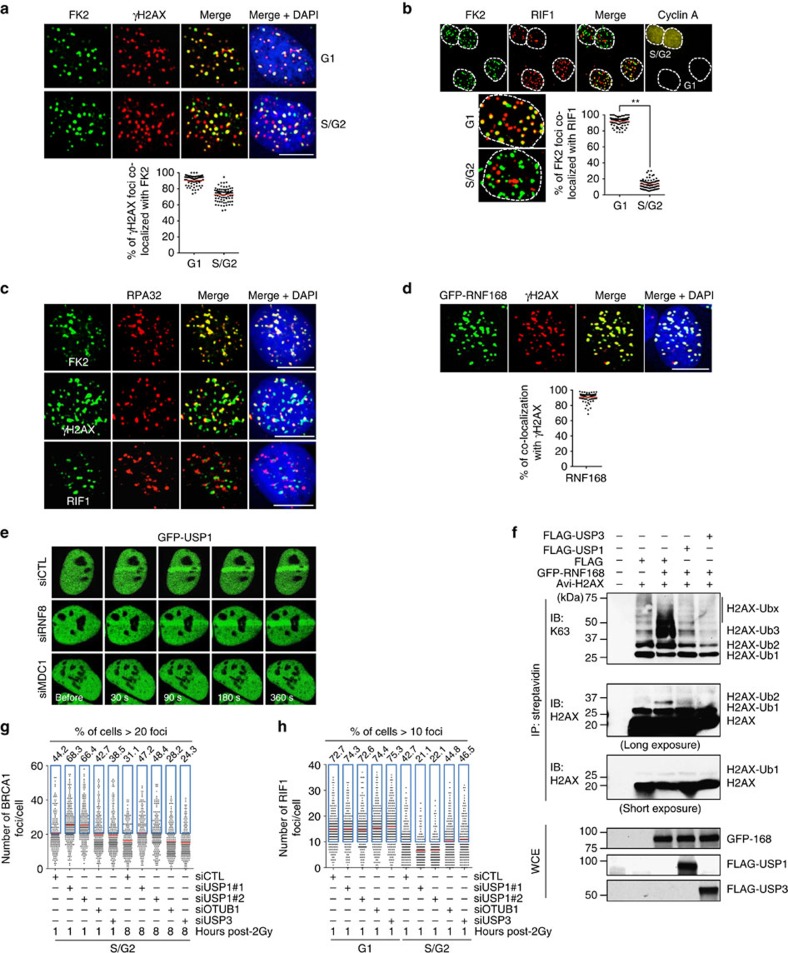
RIF1 IRIF in S/G2 cells lack poly-ubiquitination modification. (**a**) Co-staining of FK2 with γH2AX in U2OS cells 1 h after 2 Gy IR. The dot plot graph shows the percentages of γH2AX co-localization in the cells (*n*>78). (**b**) Co-staining of FK2, RIF1 and cyclin A in U2OS cells 1 h after 2 Gy IR. Enlarged G1 and S/G2 images of FK2 and RIF1 co-staining are from the merged image in upper panel, and the graph shows the percentages of FK2 foci positive for RIF1 in G1 and S/G2 cells (*n*>87). (**c**) Co-staining of FK2, γH2AX or RIF1 with RPA32 in U2OS cells. After 2 Gy IR, the cells were pre-extracted before immunofluorescence staining of the indicated proteins. (**d**) Co-localization of GFP-tagged RNF168 with γH2AX. U2OS cells transfected with GFP-tagged RNF168 expression plasmid were irradiated with 2 Gy and harvested 1 h later for analysis. The dot plot graph shows the percentages of γH2AX co-localization with RNF168 in the cells (*n*>78). (**e**) Mobilization of USP1 to sites of DNA damage. U2OS cells transfected with GFP-USP1 and the indicated siRNAs were microirradiated with laser, and imaged. (**f**) *In vivo* ubiquitination of H2AX. HeLa cells (stably expressing NLS-BirA and Avi-tagged H2AX or NLS-BirA alone) were co-transfected with GFP-RNF168 and FLAG-USP1 or FLAG-USP3, irradiated and analysed. (**g**,**h**) Quantification of BRCA1 (>170 cells analysed) (**g**) and RIF1 (>190 cells analysed) (**h**) focus number in U2OS-Fucci cells transfected with the indicated siRNAs. Blue box designates the cells with more than 20 (BRCA1 positive) and 10 (RIF1 positive) foci, respectively. The percentages of BRCA1-positive and RIF1-positive cells are indicated above each box. ** (in **b**) indicates *P*<0.001 (student’s *t*-test). Scale bars represent 10 μm.

The finding that depleting RIF1, an antagonist of BRCA1, could not increase the number of BRCA1 IRIF suggests that either Pro-NHEJ sites contain an inhibitor(s) of BRCA1 recruitment or they lack poly-ubiquitination modification (mainly K63-linked). To test which possibility is more likely, we first co-stained γH2AX (to mark most if not all DSBs) and poly-ubiquitin with FK2 antibodies. As shown in Fig. 2a, while more than 90% γH2AX foci were co-stained with FK2, not all γH2AX foci were positive for FK2 in S/G2, only 70% (±8.05%) on average (with a range of 47.5 to 87.5%). Further, while more than 90% (±6.13%) of FK2 foci co-stained with RIF1 in G1 cells, the co-localization diminished in S/G2 cells (Fig. 2b). Thus, the reason that Pro-NHEJ sites cannot recruit BRCA1, even in the absence of RIF1, is most likely the lack of poly-ubiquitin modification.

A necessary step in HDR is to resect the broken ends and generate ssDNA which is bound by RPA (replication protein A)^38^. The bound RPA then initiates a series of events including activation of ATR, Chk1, and the recruitment of BRCA1, BRCA2 and RAD51 (ref. 39). Therefore, RPA-decorated DSBs are intended for HDR or Pro-HDR, which implies co-localization of RPA IRIF with poly-ubiquitin chains. Indeed, co-staining of RPA and poly-ubiquitin demonstrated overlapping between the two. As expected, RPA only partially overlapped with γH2AX foci (Fig. 2c), and RPA and RIF1 IRIF do not overlap (Fig. 2c).

These results indicate that DSBs in S/G2 cells are processed into two different kinds, one that possesses poly-Ub chains and is competent for BRCA1 recruitment (thus Pro-HDR), and another that does not and is incompetent for BRCA1 recruitment (but competent for RIF1, thus Pro-NHEJ). Based on the percentage of FK2-positive γH2AX foci (Fig. 2c), the ratio of Pro-HDR sites over Pro-NHEJ sites in a given cell was calculated to be 0.90 to 7.0, with an average of 2.33, indicating that HDR prevails in S/G2 cells. However, this range of ratios was obtained with U2OS cells. It may change in different types of cells.

### USP1 prevents the accumulation of poly-Ub modification

The reason that the Pro-NHEJ DSB sites do not have poly-ubiquitination modification may be two-fold. One is that it lacks the E3 ligase RNF168 and another that the poly-Ub chains generated are actively destroyed *in situ* by a deubiquitinating enzyme (DUB). We therefore looked for evidence that would support either of these two possibilities. Co-staining of γH2AX with RNF168 showed that the two overlapped more than 90% (Fig. 2d), suggesting that the first possibility is unlikely, which is consistent with the notion that RNF168 is also required for 53BP1 recruitment^25^. For the second possibility, there are several DUBs including OTUB1, USP3, USP44 and POH1 that were reported to be able to remove K63-linked Ub at damage sites when ectopically expressed^40^,^41^,^42^,^43^, but apparently, none of them could do it at their endogenous levels, otherwise there would be no K63-linked Ub present at DSBs at all. Thus, we thought that such a DUB must be recruited to DNA damage sites. To that end, we found USP1 was mobilized to DNA damage sites in an MDC1-dependent, but RNF8-independent manner (Fig. 2e). Since the mobilization of MDC1 depends on ATM, the mobilization of USP1 also depends on ATM as expected (Supplementary Fig. 2a). However, USP3 and OTUB1, both reported to be able to inhibit H2A(X) ubiquitination^40^,^41^, did not show mobilization following microirradiation (Supplementary Fig. 2b). We further confirmed the recruitment of endogenous USP1 to microirradiated regions using USP1 specific antibodies (Supplementary Fig. 2c). We also found that the formation of K63-linked poly-ubiquitin chains at DNA damage sites was dramatically suppressed by ectopic expression of USP1 (Supplementary Fig. 2d) and such a suppression depended on its deubiquitinase activity as the expression of the catalytically inactive USP1 (C90S) had no effect (Supplementary Fig. 2e). In agreement with previous reports^40^,^44^, overexpression of OTUB1 and USP3 also highly inhibited K63 linkage formation following microirradiation (Supplementary Fig. 2f). In addition, we observed a marked reduction of H2AX poly-ubiquitination in cells overexpressing USP1 in an *in vivo* ubiquitination assay (Fig. 2f). USP1 at DSB sites very likely also removes K63-linked poly-Ubs formed on H1 (ref. 45) or K27-linked Poly-Ubs^45^ as well to block BRCA1 recruitment. Indeed, ectopic expression of USP1 significantly inhibited BRCA1, but increased RIF1 focus formation in S/G2 cells (Supplementary Fig. 2g,h).

Unlike OTUB1 and USP3 which inhibited both BRCA1 and 53BP1 focus formation when overexpressed^40^,^41^, 53BP1 focus formation remained largely unaffected when USP1 was overexpressed (Supplementary Fig. 2i). In contrast to the overexpression, the depletion of USP1 increased the number of BRCA1 foci but decreased the number of RIF1 foci in S/G2 cells (Fig. 2g,h, Supplementary Fig. 2j,k). However, the depletion of either OTUB1 or USP3 had no significant effects on the focus formation of either BRCA1 or RIF1 in S/G2 cells (Fig. 2g,h, Supplementary Fig. 2j,k). These results suggest that USP1 is an inhibitor of DSB-induced chromatin ubiquitination in S/G2 cells. Given that the deubiquitinase activity of USP1 is highly regulated by its interacting partner, UAF1 (ref. 46), we found that the depletion of UAF1 with two distinct siRNAs also resulted in an increase of BRCA1 foci (Supplementary Fig. 2l), and at the same time a decrease of RIF1 foci in S/G2 cells (Supplementary Fig. 2m).

### The anaphase-promoting complex functions at DNA damage sites

**Figure 3:**
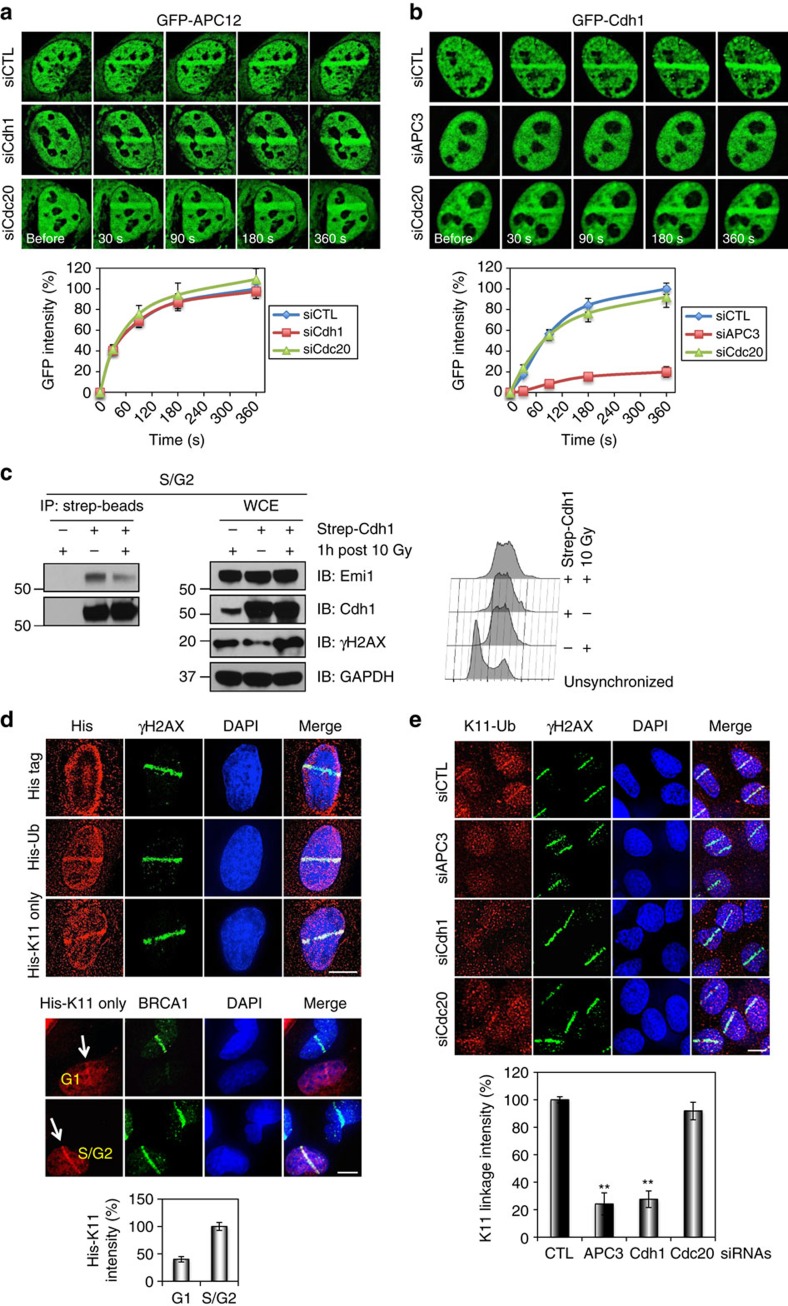
The anaphase promoting complex protects poly-ubiquitin signalling at DSBs. (**a**,**b**) Mobilization of APC12 (**a**) and Cdh1 (**b**) to sites of DNA damage. U2OS cells were transfected with GFP-APC12 or GFP-Cdh1 expression plasmids and the indicated siRNAs, microirradiated with laser and imaged at different time points. (**c**) Interaction of Cdh1 with Emi1 in S/G2 cells. HeLa cells stably expressing strep-tagged Cdh1 were synchronized with double thymidine, released into S/G2, exposed to 10 Gy IR and collected after 1 h recovery for immunoprecipitation analysis. (**d**) The accumulation of K11-linked poly-ubiquitin at sites of DNA damage. U2OS cells were transfected with ubiquitin (Ub) constructs, microirradiated and processed 30 min later for immunostaining of His tag and BRCA1. (**e**) The accumulation of the endogenous K11-linked Ub chains at sites of DNA damage. Immunostaining of K11-linked Ub and γH2AX was performed 30 min after microirradiation. Top: representative images of K11-linked poly-(Ub) and γH2AX immunostaining in U2OS cells transfected with the indicated siRNAs. Bottom: quantification of K11 linkage intensity at microirradiated regions (>110 cells quantificated). Error bars indicate SEM from three independent experiments. ** (in **e**) indicates *P*<0.001 (student’s *t*-test). Scale bars represent 10 μm.

Since both Pro-HDR and Pro-NHEJ DSB sites co-exist in a single S/G2 cell, how then does the cell select which site to recruit USP1 (to erase poly-ubiquitination) and which site not to? Alternatively, a cell can make the selection by destroying USP1 at some sites but not at others. The latter seemed more attractive as we knew that USP1 was a substrate of APC/C^8^. Specifically, USP1 is brought to APC for ubiquitination by one of its two adaptor proteins Cdh1 (Fzr1). We reasoned that if USP1 was destroyed by APC^Cdh1^ at some DSB sites, this E3 complex itself must be able to go to DBS sites as well. Indeed, we found that APC and Cdh1 could be mobilized to DNA damage sites, and the mobilization was MDC1-dependent, but RNF8-independent (Supplementary Fig. 3a,b). We also confirmed the recruitment of endogenous APC12 to microirradiated regions using APC12 specific antibodies (Supplementary Fig. 3c). Unfortunately, we were not successful to find Cdh1 antibodies suitable for immunofluorescent staining. RNF8 relocalization to sites of DNA damage was affected by neither Cdh1 nor APC3 depletion, indicating that RNF8 and APC^Cdh1^ complex localize to DSBs independent of each other (Supplementary Fig. 3d). It was shown previously that Cdc27, a core subunit of APC, could be phosphorylated by ATM, which creates a phosphorylated motif at the C-terminus recognizable by MDC1 (ref. 47). Thus, it is very likely that in response to DNA damage, Cdc27 is phosphorylated by ATM and the whole APC complex (with or without its adaptors) is then recruited to damage sites via the interaction between Cdc27 and MDC1. Consistent with that, Cdh1 depends on APC to be recruited, while APC recruitment does not require Cdh1 (Fig. 3a,b). Cdc20, another activator of APC, is required for neither APC nor Cdh1 recruitment to DSBs (Fig. 3a,b).

APC^Cdh1^ is active in G1 but is inactivated in S/G2 through interaction with the inhibitor Emi1 and the phosphorylation of Cdh1 by cyclin-dependent kinases^12^,^48^. However, it has been previously reported that APC^Cdh1^ could be reactivated in response to DNA damage in S/G2 phase cells as evidenced by the increased binding of Cdh1 to APC^10^,^49^, the activity of purified APC/C from irradiated G2 cells in *in vitro* ubiquitination assays^49^, a shift of Cdh1 towards the less phosphorylated state following IR treatment in 2D gel analysis^21^, and a reduction of the expression of Emi1 induced by p53 and p21 (ref. 50). It is also possible that DNA damage may interrupt the interaction between Emi1 and APC^Cdh1^. To that end, we analysed the binding of Cdh1 to Emi1. As expected, the binding between the two is significantly reduced following DNA damage in S/G2 phase cells (Fig. 3c).

It is known that APC catalyses the formation of K11-linked poly-Ub chains on its substrates^6^. To see if APC^Cdh1^ recruited to DNA damage sites is functional, we first determined whether APC-specific E2s were also recruited to damage sites. As shown in Supplementary Fig. 3e, both UBE2C and UBE2S could localize to microirradiated sites. Next, we stained for the presence of K11-linked Ub. As shown in Fig. 3d,e, K11-linked Ub could be detected at damage sites either through staining of ectopically expressed tagged K11-only ubiquitin or through K11-linked Ub-specific antibodies. Interestingly, K11-linked Ub chains were accumulated to a much less extent at DNA damage sites in G1 than in S/G2 cells (Fig. 3d). Significantly, the K11-linked Ub signal diminished in APC3 as well as in Cdh1 depleted cells (Fig. 3e). These data suggest that APC^Cdh1^ is a novel DNA damage response E3 ligase functioning in an RNF8-independent manner and promotes the formation of K11-linked Ub chains at DNA damage sites primarily in S/G2 cells.

### USP1 is targeted by APC^Cdh1^ at DSBs

**Figure 4:**
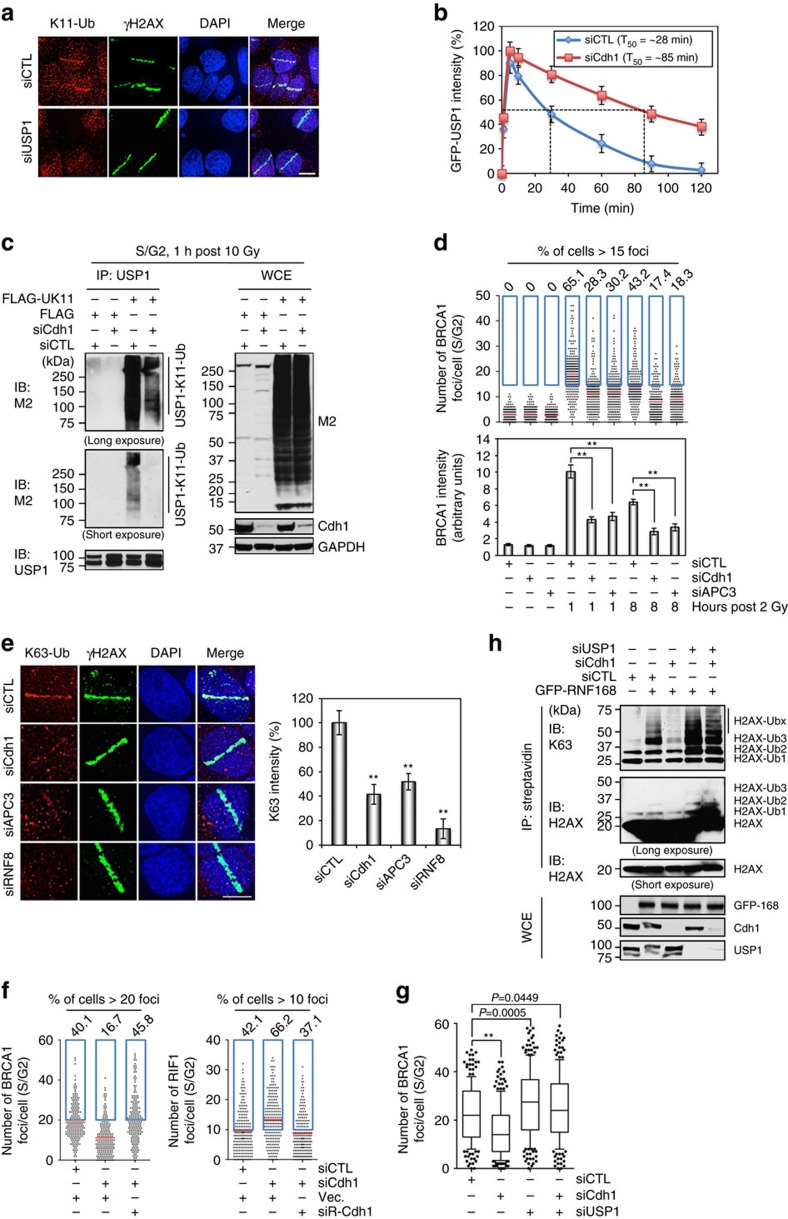
USP1 is targeted by APC^Cdh1^ at DSBs. (**a**) The K11-linked ubiquitin chains were primarily formed on USP1. U2OS cells transfected with control or USP1 siRNA were microirradiated and processed for immunostaining 30 min later. (**b**) The mobilization kinetics of GFP-USP1 to sites of DNA damage. GFP-USP1 was expressed in U2OS cells transfected with the indicated siRNAs and its mobilization was monitored for 2 h following the microirradiation. (**c**) *In vivo* ubiquitination assay of endogenous USP1 in the presence or absence of Cdh1 after DNA damage. Cell lysates were prepared as described in methods. (**d**) Quantification of BRCA1 focus number and intensity in Cdh1- and APC3-depleted cells. The cells were subjected to 2 Gy IR 3 days after the siRNA transfection. Top panel: quantification of BRCA1 foci per cell. Blue box designates the cells with more than 15 foci, whose percentage is indicated above each box. Bottom panel: average intensities of BRCA1 focus per cell (*n*>195). (**e**) The accumulation of endogenous K63-linked ubiquitin chains at damage sites. U2OS cells were microirradiated and recovered for 30 min before the immunofluorescence analysis of K63-linked Ub and γH2AX. Left panel: representative images of K63-linked Ub and γH2AX. Right panel: quantification of K63-linked Ub at microirradiated regions (*n*>102). (**f**) Quantification of BRCA1 (*n*>191) and RIF1 (*n*>197) focus formation in U2OS-Fucci cells transfected with the indicated siRNAs and siCdh1-resistant Cdh1 constructs. Blue box designates the cells with more than 20 (BRCA1 positive) and 10 (RIF1 positive) foci, respectively. The percentages of BRCA1-positive and RIF1-positive cells are indicated above each box. (**g**) Quantification of BRCA1 focus number in the cells transfected with the indicated siRNAs (*n*>200). (**h**) *In vivo* ubiquitination of H2AX in Cdh1-, USP1- or both Cdh1- and USP1-depleted cells. The indicated siRNAs were transfected first, followed by GFP-RNF168 transfection 48 h later, and another 24 h later, the cells were irradiated with 10 Gy and harvested 1 h after IR for the analysis of H2AX ubiquitination. Error bars indicate SEM from three independent experiments. ** indicates *P*<0.001 (student’s *t*-test). Scale bars represent 10 μm.

If USP1 were the target of APC^Cdh1^ at damage sites, one would see the intensity of K11-linked Ub signal diminishing in USP1-depleted cells. Indeed, as shown in Fig. 4a, the K11-linked Ub signal was close to undetectable in USP1 knockdown cells, suggesting that USP1 is the major, if not the only, substrate of APC^Cdh1^ at DNA damage sites. In agreement with that, the depletion of Cdh1 markedly prolonged the retention of USP1 at DNA damage sites (Fig. 4b and Supplementary Fig. 4a).

A previous report has shown that USP1 is ubiquitinated by APC^Cdh1^ in an *in vitro* ubiquitination assay^8^. Using K11-only mutant ubiquitin, we performed an *in vivo* ubiquitination assay of USP1 in S/G2 cells depleted of Cdh1. Figure 4c shows that USP1 was ubiquitinated (K11-linked) *in vivo*. Although the depletion of Cdh1 increased USP1 expression, the levels of ubiquitinated USP1 were significantly reduced, suggesting that the formation of K11 linkage on USP1 was highly dependent on the function of Cdh1.

Given that USP1 suppresses BRCA1, we expect that compromising APC function would also lead to an apparent suppression on BRCA1 focus formation. Indeed, the depletion of either APC3 or Cdh1 caused a severe reduction in the number and the intensity of BRCA1 foci as well as a similar decrease in K63-linked Ub signal strength at micro-irradiated regions (Fig. 4d,e, and Supplementary Fig. 4b). The depletion of Cdh1 via shRNA targeting a different region of Cdh1 sequence also resulted in a reduction of BRCA1 and Rad51 foci as well as a reduction of the K63-linked poly-(Ub) at DNA damage sites (Supplementary Fig. 4c). Furthermore, both BRCA1 and RIF1 focus formation were restored to normal by the ectopic expression of siRNA-resistant Cdh1 in the cells depleted of the adaptor (Fig. 4f and Supplementary Fig. 4d,e,f), indicating that the effects of Cdh1 depletion on BRCA1 and RIF1 focus formation we observed were not mediated by off-target effects of the siRNA. More importantly, the simultaneous depletion of Cdh1 and USP1 restored near completely the focus formation of BRCA1 and Rad51, as well as the accumulation of K63-linked Ub chains (Fig. 4g and Supplementary Fig. 4g,h,i). Consistent with these immunostaining results, we observed a significant decrease of H2AX ubiquitination in Cdh1-depleted cells, but an increase in USP1-depleted cells (Fig. 4h).

**Figure 5:**
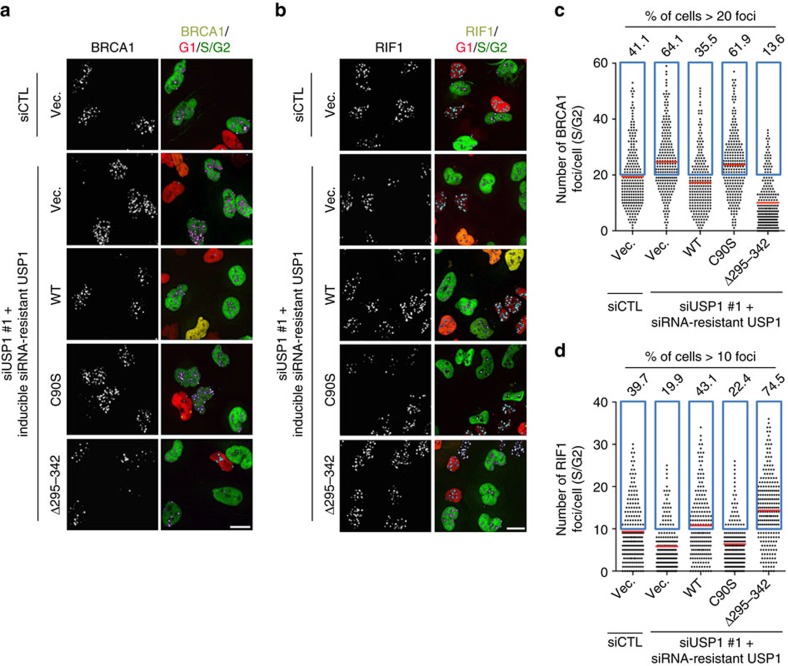
Antagonizing USP1 by APC^Cdh1^ at DNA damage sites is critical for BRCA1 recruitment. (**a**,**b**). Representative images of BRCA1 (**a**) and RIF1 (**b**) IRIF in U2OS-Fucci cells transfected with the indicated siRNAs and expression vectors. The cells were irradiated with 2 Gy and recovered for 1 h before the immunostaining. (**c**,**d**) Quantification of BRCA1 (**c**, *n*>226) and RIF1 (**d**, *n*>195) focus formation in **a**,**b**. Blue box designates cells with more than 20 (BRCA1 positive) and 10 (RIF1 positive) foci, respectively. The percentages of BRCA1-positive and RIF1-positive cells are indicated above each box. Scale bars represent 10 μm.

To further establish the role of APC^Cdh1^ in antagonizing USP1 at DNA damage sites, we generated U2OS cell lines that inducibly express USP1, catalytically inactive USP1 (C90S mutant), and APC^Cdh1^-resistant USP1 (Δ295-342 mutant)^8^ at the endogenous level of USP1 expression plus a line with the expression vector only as a control (Supplementary Fig. 5). All three forms of USP1 expressed were made siUSP1 resistant. These cells were then depleted of USP1 and examined for their ability to form BRCA1 and RIF1 IRIF. As shown in Fig. 5, the depletion of USP1 in control cells (no ectopic USP1 expression) expectedly caused an increase of BRCA1 IRIF but a decrease of RIF1 IRIF (as seen in Fig. 2g,h). These effects were largely eliminated by re-expressing wild-type USP1, but not by re-expressing the catalytically inactive USP1. In sharp contrast, however, USP1 (Δ295-342)-expressing cells showed greatly diminished ability to form BRCA1 foci but at the same time showed greatly enhanced ability to form RIF1 foci (Fig. 5a–d), as if these cells were overexpressing wild-type USP1 (see Supplementary Fig. 2g,h). Thus, expression of APC^Cdh1^-resistant USP1 at endogenous levels favours the generation of Pro-NHEJ sites at the expense of Pro-HDR sites.

### Chk1-Cdc14B signalling is required for Cdh1 recruitment

**Figure 6:**
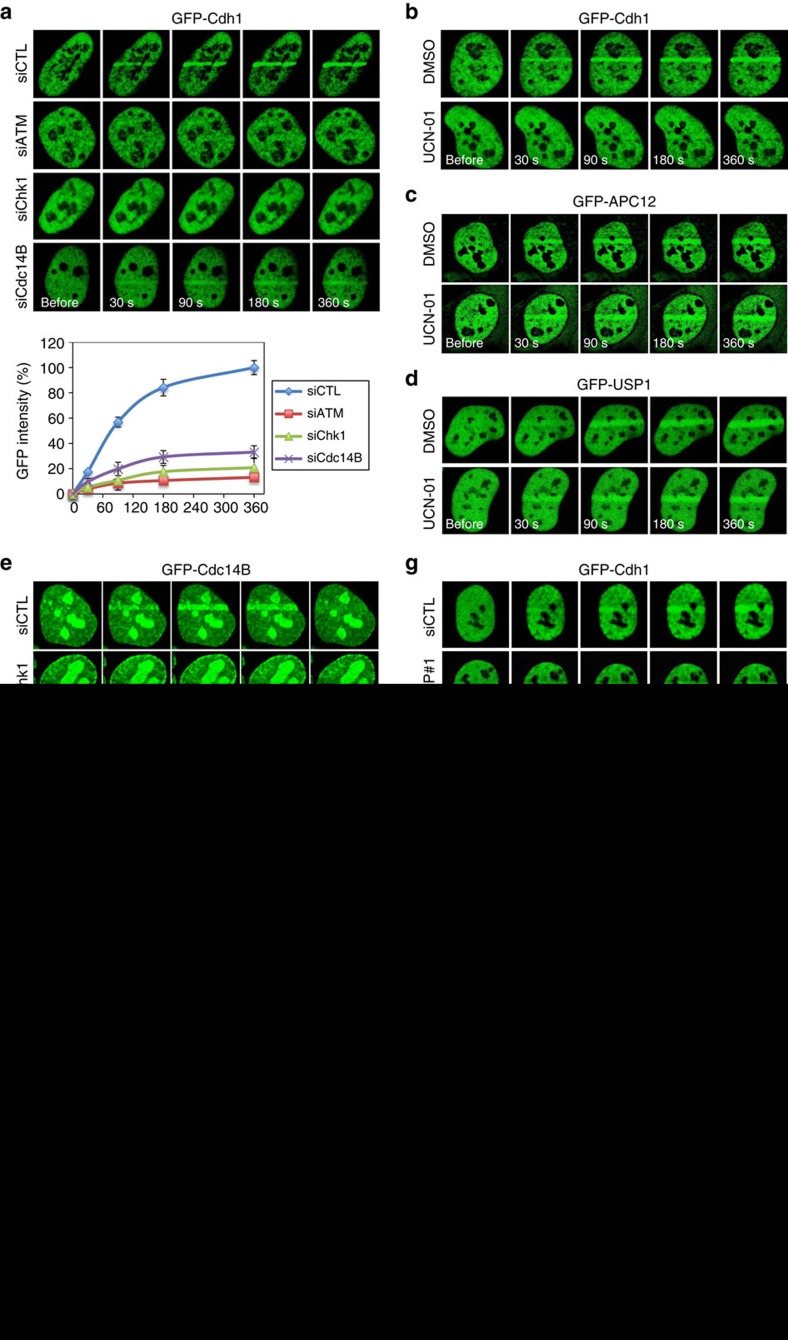
Chk1-Cdc14B signalling is required for Cdh1 recruitment. (**a**) The mobilization of Cdh1 to sites of DNA damage. U2OS were transfected with GFP-Cdh1 and the indicated siRNAs, laser microirradiated and imaged. The graph shows the intensities at the irradiated regions of GFP-Cdh1 over time. (**b**) The effect of Chk1 inhibitor UCN-01 on mobilization of Cdh1 to sites of DNA damage. Twenty-four hours after the transfection of GFP-Cdh1, the cells were treated with UCN-01 (300 nM) for another 24 h followed by microirradiation. (**c**,**d**) The effect of UCN-01 on mobilization of APC12 (**c**) and USP1 (**d**) to sites of DNA damage. The cells were similarly treated as those in **b**. (**e**) The mobilization of Cdc14B to sites of DNA damage. The experiment was performed similarly as in **a**. (**f**) The mobilization of Chk1 to sites of DNA damage. Top panel: representative images of GFP-Chk1 recruitment following microirradiation; bottom panel: quantification of GFP-Chk1 intensity at the damaged regions. The cells used were U2OS-Fucci or U2OS synchronized to S/G2 through double thymidine block and release. (**g**) The mobilization of Cdh1 to microirradiated regions is suppressed by CtIP depletion. (**h**) Quantification of IR-induced BRCA1 focus formation in U2OS-Fucci cells transfected with control and two different Chk1 siRNAs. Three days after the siRNA transfection, immunostaining was performed at 1 and 8 h post 2 Gy of IR. Left panel: representative images of BRCA1 foci. Right panel: quantification of BRCA1 focus number in the cells (*n*>174). Error bars indicate s.e.m.

Both APC complex and USP1 depend on MDC1 to be recruited to DNA damage sites (Supplementary Fig. 3a, Fig. 2e), but only the Pro-HDR sites seem to be able to activate APC and destroy USP1 (Fig. 4g). One must wonder what is present at Pro-HDR sites but absent at Pro-NHEJ sites that could activate APC, therefore protecting the poly-ubiquitin signal required for BRCA1 recruitment. We reasoned that by examining the requirement of known DDR signalling regulators in the recruitment of the APC activator Cdh1, we might unravel clues to that question. To that end, we found that Cdh1 recruitment not only depended on ATM similarly as APC complex itself (Fig. 6a), but it also required Chk1 (Fig. 6a,b). However, neither APC (APC12) (Fig. 6c) nor USP1 (Fig. 6d) required Chk1. In these experiments, ATM and Chk1 kinases was inactivated via either siRNA or chemical inhibition.

We and others have shown previously that Cdc14 lies upstream of Cdh1 in DNA damage repair, likely through dephosphorylating (hence activating) Cdh1 (refs 10, 21), and consistent with that, we found that the recruitment of Cdh1 to DNA damage sites largely depended on Cdc14B (Fig. 6a), whereas APC did not (Supplementary Fig. 6a). It has been reported that Chk1 regulates Cdc14B function by promoting its release from nucleoli^10^,^22^. Thus, it is likely that Chk1 regulates the localization of Cdh1 by releasing Cdc14B from nucleolus to nucleoplasm where the phosphatase can then act on Cdh1. Interestingly, Cdc14B is also localized to damage sites (although transiently, for about 5 min) and the localization depends on Chk1 (Fig. 6e), suggesting that Cdh1 dephosphorylation and activation may be limited to DNA damage sites where Cdc14B is present. Given that GFP-Cdc14B was already present in the nucleoplasm in our experiment due to overexpression (Fig. 6e), the function of Chk1 in the localization of Cdc14B to damage sites is unlikely just to release the phosphatase from nucleoli. It probably also actively helps the mobilization of the phosphatase to damage sites. Consistent with that, we have shown previously that Cdc14B interacts with Chk1 (ref. 22). Moreover, Chk1 itself was localized to damage sites (Fig. 6f). In agreement with the finding that Chk1 is released from damage sites soon after activation^51^,^52^, we found Chk1’s presence at damage sites peeked by 2 min which strikingly coincide with the duration of Cdc14B at damage sites (Fig. 6e), reinforcing the conclusion that Chk1 brings the phosphatase to damages sites.

Given that Chk1 is activated by ssDNA-RPA-ATR signalling in which ssDNA is the product of end resection in preparation of the DSB for HDR^39^, we asked if CtIP, a key end resection factor along with MRN complex^53^, is required for Cdh1 recruitment. Indeed, as shown in Fig. 6g, depletion of CtIP substantially reduced the amount of Cdh1 recruited to DNA damage sites. As a result, the accumulation of poly-Ubs was also reduced significantly (Supplementary Fig. 6b).

Next, we determined whether Chk1 regulated accumulation of BRCA1 at sites of DNA damage. Chk1 was depleted in U2OS-Fucci cells with two distinct siRNAs and the cells were subjected to 2 Gy γ-irradiation. As expected, BRCA1 IRIF was significantly inhibited in Chk1-depleted cells (Fig. 6h). Taken together, these results strongly suggest that there is a separate and Chk1-dependent pathway, in addition to the one that recruits APC, that regulates Cdh1 localization and activation at Pro-HDR sites. Since Chk1 is activated by ssDNA-RPA-ATR signalling in which ssDNA is the product of end resection in preparation of the DSB for HDR^39^, the requirement of Chk1 links Cdh1 recruitment to end resection, and provides additional support to the notion that Chk1 is required for homologous recombination in mammalian cells^54^.

### Cdh1 is required for HDR repair of DSBs

**Figure 7:**
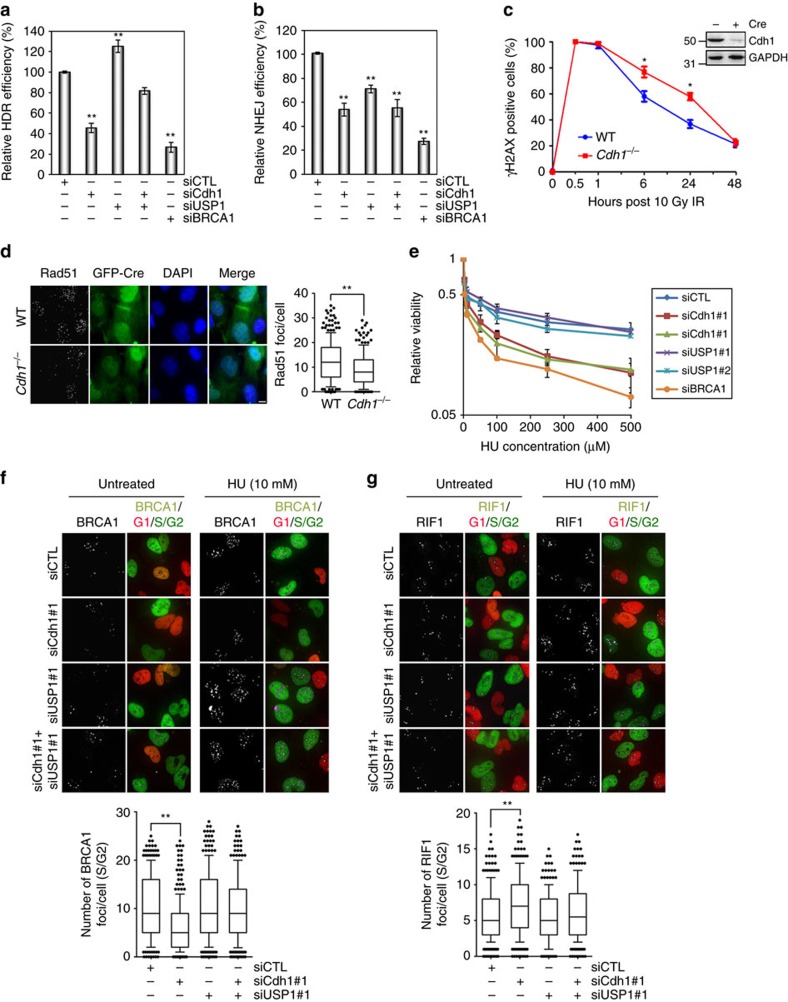
Cdh1 is required for HDR repair of DSBs. (**a**,**b**) Quantification of HDR (**a**) and NHEJ (**b**) assays in cells transfected with indicated siRNAs. The HDR (the percentage of GFP+ cells among mCherry+ cells) and NHEJ (the percentage of tdTomato+ cells among GFP+ cells) efficiencies in cells transfected with the indicated siRNAs were normalized to that in control (siCTL) cells. (**c**) Quantification of γH2AX foci in WT and *Cdh1*^−/−^ MEFs after 10 Gy IR. (**d**) Immunostaining of Rad51 in WT and *Cdh1*^−/−^ MEFs after 10 Gy IR. The cells were fixed 1 h after the irradiation, and processed for immunofluorescence analysis. (**e**) Survival of U2OS cells transfected with the indicated siRNAs after hydroxyurea treatment. Twenty four hours after transfection of the siRNAs, the cells were treated with the indicated concentrations of HU for 24 h and then cultured in fresh media for additional 2 days before the viability test. (**f**,**g**) Immunofluorescence analysis of BRCA1 (**f**) and RIF1 (**g**) in U2OS-Fucci cells transfected with the indicated siRNAs. Three days after the siRNA transfection, the cells were treated with 10 mM HU for 2 h and collected for the analysis. Top panel: representative images of BRCA1 and RIF1 foci in cells transfected with indicated siRNAs. Bottom panel: quantification of BRCA1 and RIF1 focus number per cell (*n*>218). Error bars indicate SEM. ** indicates *P*<0.001 (student’s *t*-test).

Having established that the recruitment of BRCA1 requires the function of APC^Cdh1^, we expected that the deficiency in Cdh1 would compromise HDR. We therefore examined the HDR efficiency in U2OS cells carrying a HDR reporter^21^. The depletion of Cdh1 caused a reduction in HDR close to that caused by BRCA1 depletion, but the depletion of USP1, as expected, resulted in an apparent increase in the HDR efficiency (Fig. 7a). More importantly, the depletion of USP1 restored the reduction in HDR efficiency caused by compromised Cdh1 function, reinforcing the notion that APC^Cdh1^ antagonizes USP1. Under these conditions, we did not observe significant effects exerted by Cdh1, USP1 or BRCA1 depletion (Supplementary Fig. 7a), indicating that the differences in HDR efficiencies are unlikely caused by disturbance in cell cycle phase distribution. Further, re-expression of siRNA-resistant and APC^Cdh1^-resistant USP1 in USP1-depleted U2OS cells severely blocked HDR repair (Supplementary Fig. 7b), mimicking the loss of Cdh1 (Fig. 7a). In contrast, the depletion of USP1 resulted in a decrease in the NHEJ efficiency (Fig. 7b), and the re-expression of APC^Cdh1^-resistant USP1 increased the efficiency as expected (Supplementary Fig. 7c). Interestingly, Cdh1 depletion reduced the NHEJ efficiency as well, suggesting that Cdh1 might also regulate factors involved in NHEJ repair in G1. Moreover, mouse embryonic fibroblasts (MEFs) with *Cdh1* deleted showed a delay in repairing DSBs (Fig. 7c) and defects in forming Rad51 IRIF (Fig. 7d). Taken together, these results demonstrate the importance of the interplay between APC^Cdh1^ and its substrate USP2 in the repair of DSBs.

### APC^Cdh1^ antagonizes USP1 in response to replication stress

Replication stress presents a unique problem to cells as it often results in replication fork collapse which in turn generates DSBs. These DSBs are often one-ended and can only be repaired through HDR^55^,^56^. To determine if Cdh1 plays a similar role in dealing with replication stress as with ionizing radiation, we first determined the sensitivity of the cells depleted of Cdh1 to hydroxyurea (HU) treatment. As shown in Fig. 7e, Cdh1-depleted cells displayed similar sensitivity as BRCA1-depleted cells, indicating that APC^Cdh1^ is required for HDR of DSBs resulted from collapsed replication forks. As expected, the number of BRCA1 foci reduced dramatically in Cdh1-depleted cells treated with HU, and the simultaneous depletion of USP1 with that of Cdh1 essentially restored BRCA1 focus number (Fig. 7f). Interestingly, in contrast to IR-treatment, the depletion of USP1 alone in HU-treated cells had no effect on BRCA1 foci (Fig. 7f) nor on HU sensitivity (Fig. 7e), which is consistent with the notion that DSBs resulted from replication stress are mostly dealt with HDR, not NHEJ (thus are exclusively Pro-HDR)^55^,^56^. Indeed, unlike BRCA1, RIF1 focus formation was not induced by HU treatment (Fig. 7g). However, the depletion of Cdh1 did cause an increase in RIF1 focus number in HU-treated cells, and the double depletion of Cdh1 and UPS1 turned that number back to ‘normal’ (Fig. 7g), further supporting the conclusion that there is a competition between BRCA1 and RIF1 at DSBs and the competition is swung one way or another by USP1 and APC^Cdh1^ in S/G2 cells. Moreover, compromising Chk1 function via siRNA (Supplementary Fig. 7d) or a chemical inhibitor (Supplementary Fig. 7e) also strongly inhibited HU-induced BRCA1 focus formation, consistent with Chk1’s role in recruiting and activating Cdh1.

## Discussion

We show here that DSBs in S/G2 cells are processed differentially for NHEJ or HDR. Interestingly, the ratio of the number of BRCA1 IRIF over that of RIF1 IRIF varies over a wide range among the cells (Fig. 1a), indicating that the disposition of DSBs between NHEJ and HDR in each cell is different, NHEJ dominates in some but HDR does in some others. It is known that both Ku heterodimer (for NHEJ) and MRN (MRE11, RAD50 and NBS1) complex (for HDR) can bind to the broken ends upon DNA damage, likely in a competitive fashion^57^,^58^. We speculate that the stoichiometry of Ku heterodimers and MRN complexes is different in each cell, so the observed differential disposition of DSBs.

**Figure 8:**
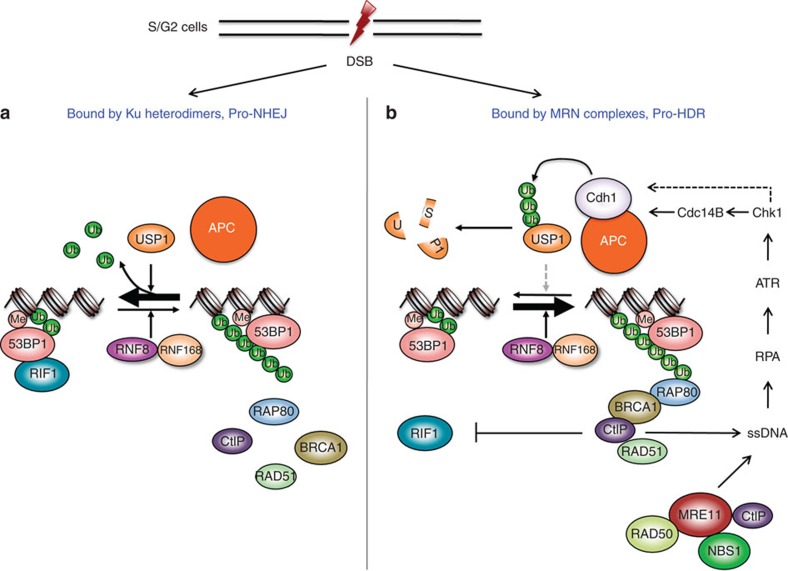
A model for the regulation of DSB repair pathway choice by USP1 and APC^Cdh1^ in S/G2 cells. The DSBs in S/G2 cells can be processed by two competing factors, the Ku heterodimers and the MRN complex. (**a**) When a DSB is bound by Ku heterodimers, the break is then destined for NHEJ. (**b**) On the other hand, when a site is bound by MRN complex, the ends are resected and ssDNA generated, leading to the activation of ATR, Chk1 and APC^Cdh1^, and eventually the destruction of USP1 and the recruitment of BRCA1. BRCA1 then expels RIF1 and reinforces the end resection, leading to further activation of ATR, Chk1 and APC.

While the broken ends are being processed either in favour of NHEJ (bound by Ku and related factors) or HDR (bound by MRN complex and related factors), ATM-γH2AX-MDC1 signalling results in the recruitment of RNF8/RNF168, USP1 and APC among others to the damage sites. RNF168 lays down K63-linked poly-Ubs but only to be destroyed by USP1, so there is no BRCA1 recruitment. However, there must be some remnants of the poly-Ub chains left (for example, see Fig. 2f, there are still some residual H2AX-2Ub) that are probably enough for 53BP1 but not for BRCA1, as the former is driven to DSB sites primarily by the attraction of methylated histones^27^,^59^. 53BP1 then brings in RIF1 and the site becomes pro-NHEJ (Fig. 8a). How then is BRCA1 recruited to make or convert those sites bound by MRN complexes for HDR? We showed that the conversion required the function of APC^Cdh1^ and we further demonstrated that the recruitment of Cdh1 depended on Cdc14B and Chk1. Thus, it is likely that MRN complexes along with its associated factors including CtIP bind to the broken ends and initiate end resection, producing ssDNA that activates ATR first then Chk1, which leads to the activation of APC and the destruction of USP1. The latter then allows the accumulation of K63-linked poly-Ub chains and the recruitment of BRCA1. The recruited BRCA1 expels RIF1 on one hand and stimulates end resection further on the other, which reinforces the activation of ATR, Chk1 and APC, making the choice of HDR irreversible (Fig. 8b). In the event of replication stress, the collapsed replication forks already contain ssDNA to begin with which activates ATR and Chk1, committing the damage for HDR. However, APC^Cdh1^ is still needed to keep USP1 (and RIF1) out, thus the sensitivity of Cdh1-depleted cells to HU treatment (Fig. 7d).

There have been several reports indicating that Chk1 is essential in homologous recombination repair but the reason is not entirely clear^54^,^60^,^61^. We believe that part of the reason is that Chk1 is required to recruit Cdh1 (Fig. 6a). This requirement, at least in part, is mediated by activating Cdc14B and bringing the phosphatase to damage sites, although we could not eliminate the possibility that Chk1 might also directly activate Cdh1 at DSB sites (Fig. 8b, dashed arrow).

APC^Cdh1^ targets a long list of proteins including oncogene products for degradation. Thus, Cdh1 has been proposed as a tumour suppressor^62^. Our data reported clearly show that it also plays an important role in DSB repair, suggesting that its role in the maintenance of genome stability contributes to its function as a potential tumour suppressor.

## Methods

### Cell culture and mice

U2OS and U2OS-Fucci cells were cultured in McCoy’s 5A medium (Sigma). HEK293T, HeLa, HeLa-Fucci and MEF cells were grown in DMEM (Sigma). All culture media were supplemented with 10% FBS and 100 units ml^−1^ penicillin and 100 μg ml^−1^ streptomycin, except the medium for MEFs that was supplemented with 15% FBS. HEK293T and HeLa cells were obtained from ATCC. U2OS-Fucci cell line was a gift from Dr Hisao Masai (Tokyo Metropolitan Institute of Medical Science) and HeLa-Fucci cell line was a gift from Dr Daniel Durocher (University of Toronto).

The Cdh1 conditional knockout mice were reported previously^63^ and crossed with a transgenic line ubiquitously expressing ER-Cre. Male and female mice at 8 weeks of age were mated and the pregnant females were killed to obtain mouse embryonic fibroblasts (MEFs) as we have done before^21^,^64^. Homozygous Cdh1 conditional MEFs were infected with GFP- or Cre-expressing adenoviruses to delete Cdh1 for experiments. The mice were in C57/BL6 background. All animal procedures were approved by the IACUC of Baylor College of Medicine.

### DNA constructs

All GFP-tagged constructs for human Cdh1, deletion Cdh1 mutants, APC12, RNF8, RNF168, UBE2C, UBE2S, USP1 and OTUB1 were generated by PCR and subcloned into the pEGFP-N1 vector (Clontech). GFP-USP3 construct was a gift from Dr Elisabetta Citterio (Netherlands Cancer Institute). All ubiquitin constructs including WT and UK11 were subcloned into pcDNA3 vector (life technologies) with either 3X FLAG or His tag as indicated. All point and deletion mutations in tumour-associated Cdh1 mutants and in USP1 mutants were introduced by site-directed mutagenesis (Agilent) using pEGFP-N1-Cdh1 or pEGFP-N1-USP1 plasmids as templates. siRNA-resistant Cdh1 and USP1 mutants were also generated by site-directed mutagenesis using pcDNA3-Flag-Cdh1 or pcDNA3-Flag-USP1 plasmids as templates. To generate inducible USP1 expression constructs, USP1 (or its variants) coding region was first subcloned into the pENTRY vector and then transferred to pINDUCER20 via Gateway technology (life technology), using standard methods^21^.

### RNA interference

All short interfering RNA (siRNA) duplexes employed in this study were from Sigma. The sequences of siRNAs targeting Cdh1, USP1, BRCA1, RIF1, 53BP1, RNF8, APC3, Chk1, OTUB1, UAF1, Cdc20, CtIP and ATM are as follows: Cdh1 #1 5′-UGAGAAGUCUCCCAGUCAGdTdT-3′, Cdh1 #2 5′-GGAUUAACGAGAAUGAGAAdTdT-3′, USP1 #1 5′-AACCCUAUGUAUGAAGGAUAUdTdT – 3′, USP1 #2 5′ – CGGCAAUACUUGCUAUCUUAdTdT-3′, BRCA1 #1 5′-GGGAUACCAUGCAACAUAAdTdT-3′, BRCA1 #2 5′-CAGCUACCCUUCCAUCAUAdTdT-3′, RIF1 #1 5′-GCATTGACTTCTCACCATAdTdT-3′, RIF1 #2 5′-CGUAGAGAUUAGUGAAACAdTdT-3′, 53BP1 5′-GAAGGACGGAGUACUAAUAdTdT-3, RNF8 5′-CAGAGAAGCUUACAGAUGUUUdTdT-3′, APC3 5′-CAAAAGAGCCUUAGUUUAAdTdT-3′, Chk1 #1 5′-GAAGCAGUCGCAGUGAAGAdTdT-3′, Chk1 #2 5′-GGGAUAUUAAACCAGAAAAdTdT-3′, OTUB1 5′-GCAAGUUCUUCGAGCACUUdTdT-3′, USP3 5′-GGAGUUAAGGAAUGGGAAAdTdT-3′, UAF1 #1 5′-GAGAUGAAGUGGAGAAGUdTdT-3′, UAF1 #2 5′-CAGCAGAGAUGUAUAGCAAdTdT-3′, Cdc20 5′-CGAAAUGACUAUUACCUGAdTdT-3′, CtIP #1 5′-GCUAAAACAGGAACGAAUCdTdT-3′, CtIP #2 5′-UCCACAACAUAAUCCUAAUdTdT-3′, and ATM 5′-CGCAUGUGAUUAAAGCAACdTdT-3′. siRNAs targeting MDC1 and Cdc14B were previously described^21^,^65^. Both plasmid and siRNA transfection were carried out using Lipofectamine 2000 (Life technologies) following the manufacturer’s protocol. Cells treated with siRNAs were used for further analyses within 72 h after transfection.

### Antibodies and reagents

The following antibodies were used in this study: anti-His-tag (sc-803, 1:1,000), anti-RNF8 (sc-271462, 1:1,000), anti-APC3 (sc-13154, 1:1,000), anti-BRCA1 (sc-6954, 1:80), anti-cyclin A (sc-751, 1:200), anti-Emi1 (sc-365212, 1:200 (IF), 1:1,000 (WB)), anti-Cdh1 (sc-56312, 1:200 (IF), 1:1,000 (WB)) (Santa Cruz Biotechnology); anti-γH2AX (05-636, 1:250 (IF), 1:1,000 (WB)), anti-ubiquitin K11 linkage (MABS107-I, 1:200), anti-ubiquitin K63 linkage (05-1308, 1:200 (IF), 1:1,000 (WB)), anti-GAPDH (MAB374, 1:5,000) (Millipore); anti-APC12 (13559-1-AP, 1:100), anti-USP1 (14346-1-AP, 1:200 (IF), 1:1,000 (WB)) (Proteintech); anti-FK2 (BML-PW8810-0100, 1:250) (Enzo Life Sciences); anti-RAP80 (NBP1-87156, 1:250), anti-RAD51 (NB100-148, 1:250), anti-53BP1 (NB100-304, 1:250), anti-DNA-PKcs (phosphoT2609, 1:200) (NB100-2077) (Novus); anti-CtIP (61141, 1:250) (Active Motif); anti-RIF1 (A300-567A, 1:200) (Bethyl Laboratory); anti-MDC1 (ab114143, 1:1,000), anti-H2AX (ab11175, 1:2,000) (Abcam); anti-RPA32 (2208S, 1:250) (Cell Signaling); and anti-FLAG tag (F1804, 1:1,000) (Sigma). KU-55933 was obtained from Santa Cruz Biotechnology. UCN-01 and caffeine were purchased from Sigma.

### Western blotting

The cells were collected after treatments and lysed with lysis buffer (50 mM Tris-HCl, 150 mM NaCl, 1% Triton X-100 and 0.1% SDS). SDS loading buffer was added to samples. After boiling, samples were loaded on SDS–PAGE gels and transferred to PVDF membranes. Membranes were probed with indicated antibodies and detection was performed by chemiluminescent immunoassay. Uncropped gel images can be found in Supplementary Fig. 8.

### *In vivo* ubiquitination assay

After transfection of the indicated constructs or siRNAs, HeLa cells stably expressing NLS-BirA and Avi-H2AX were treated with 2 μg ml^−1^ of biotin (Sigma) overnight, and then collected 1 h after 10 Gy IR. For endogenous USP1 ubiquitination assay or the interaction between Cdh1 and Emi1, HeLa cells were transfected with the indicated siRNAs and various expression constructs. The transfected cells were synchronized with double thymidine block and released into fresh media for 6–8 h when they had reached S/G2 (the cell cycle progression was monitored with FACS analysis). In USP1 ubiquitination experiment, MG132 (10 μM) was added for 6 h during the release. The cells were then irradiated with 10 Gy and collected 1 h after the irradiation. The collected cells were lysed in 100 mM Tris-HCl, pH 8.0, 200 mM NaCl, 6 M Urea and 0.5% SDS (lysis buffer) plus phosphatase (GenDEPOT) and protease inhibitors (Roche) as well as 50 μM PR-619 (a pan-DUB inhibitor, Millipore). After sonication, the supernatants were diluted in lysis buffer without SDS and incubated with Streptavidin-Agarose Slurry (Thermo Scientific) or USP1 antibody-conjugated to protein G beads for 6 h at 4 °C. The agarose beads were then spun down, washed with the lysis buffer (without SDS), and prepared for Western blot analysis.

### Quantitative RT–PCR

Total RNA was extracted using RNeasy mini kit (Qiagen) and cDNA was prepared using RT reagent kit (Takara). quantitative PCR with reverse transcription was performed on a StepOne Plus Real-Time PCR system (Applied Biosystems) using FastStart Universal SYBR Green master mix (Roche). Expression of GAPDH was used to normalize relative expression of Cdc14b mRNA.

### Laser microirradiation

The cells were plated on 35 mm glass bottom dishes (MatTek Corporation) overnight. Twenty-four hours after transfection with the indicated constructs, the cells were treated with 1 μg ml^−1^ of Hoechst 33258 (Sigma) for 2 h and DNA damages were introduced by micro-irradiation using Nikon A1R-s inverted confocal microscope (Nikon, Inc.) equipped with a 405 nm diode laser focused through a × 60 Plan Apo/1.4 NA Oil objective and a 37 °C heating chamber. Time-lapse imaging for the recruitment of EGFP- and RFP-tagged proteins to sites of DNA damage was recorded after excitation with 488 and 561 nm lasers, respectively. For the recruitment of fluorescence-tagged proteins in the presence of chemical inhibitors, cells were treated with indicated inhibitors for 24 h on the second day of the transfection. For live cell imaging in siRNA-treated cells, the indicated constructs were transfected 48 h after the siRNA transfection.

### HDR and NHEJ assays

Briefly, U2OS cells carrying the HR repair reporter DR-GFP were transfected with pCBA-I-SceI or pCAGGS (mock transfection) 48 h after the transfection of the indicated siRNAs. pCAGGS-mCherry was co-transfected as a control for transfection efficiency. Forty-eight hours later, flow cytometry analysis was performed to detect GFP and mCherry-positive cells using LSR Fortessa (BD Biosciences). For NHEJ assay, U2OS cells were transfected with the indicated siRNAs for 72 h, and then cells were electroporated with 5 μg of linearized pCSCMV:tdTomato and 5 μg of pEGFP-N1 per 10^6^ cells. After 5 h culture, the cells were collected for flow cytometry analysis. The data were analysed with BD FACSDiva software.

### Immunofluorescence microscopy

The cells were cultured on coverslips overnight, and transfected with indicated siRNAs. Seventy-two hours after transfection, the cells were treated with gamma irradiation (2 Gy) and collected to visualize the IRIF. For pre-extraction, the cells were washed with PBS and incubated with pre-extraction buffer (0.2% Triton X-100 and 300 mM sucrose) for 2 min on ice. Then, the cells were fixed with 4% paraformaldehyde for 15 min at room temperature, and permeabilized with 0.5% Triton X-100 for 15 min. Samples were blocked with 5% goat serum and incubated with primary antibodies for 1 h at 37 °C. After washing with PBS three times, the samples were incubated with secondary antibodies for 1 h at 37 °C. DNA was visualized with DAPI. Images were acquired on Deltavision LIVE high-resolution deconvolution microscope (Applied Biosystems). For the quantification of focus number and intensity, deconvoluted images were analysed using softWoRx Explorer (Applied Precisions), CellProfiler (Broad Institute) and Prism 6 (GraphPad). The profile plots of fluorescence intensities were generated using ImageJ. *P* values were calculated with a student’s *t*-test by Prism 6 and data were considered statistically significant for *P* values<0.05.

### Cell viability assay

U2OS cells were transfected with the indicated siRNAs for 48 h, split into a 96-well plate, and treated with different concentrations of hydroxyurea for 24 h. After washing with PBS, the cells were cultured in fresh media for additional 2 days, and incubated with 0.5 mg ml^−1^ MTT for 4 h. A solubilization solution (10% SDS/0.01 M HCl) was added into each well and the absorbance at 595 nm of the samples was measured using a microplate reader.

### Data availability

All relevant data are available from the authors.

## Additional information

**How to cite this article:** Ha, K. *et al*. The anaphase promoting complex impacts repair choice by protecting ubiquitin signalling at DNA damage sites. *Nat. Commun.* **8,** 15751 doi: 10.1038/ncomms15751 (2017).

**Publisher’s note:** Springer Nature remains neutral with regard to jurisdictional claims in published maps and institutional affiliations.

## Supplementary Material

Supplementary Information Supplementary Figures
