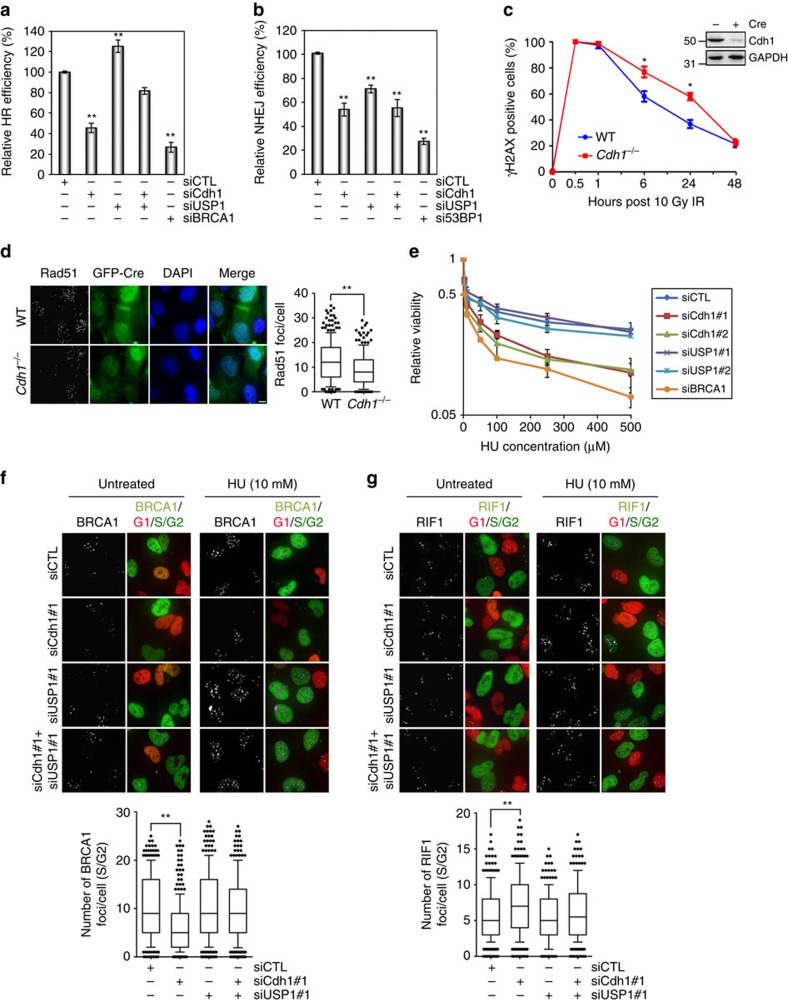# Erratum: The anaphase promoting complex impacts repair choice by protecting ubiquitin signalling at DNA damage sites

**DOI:** 10.1038/ncomms16156

**Published:** 2017-11-29

**Authors:** Kyungsoo Ha, Chengxian Ma, Han Lin, Lichun Tang, Zhusheng Lian, Fang Zhao, Ju-Mei Li, Bei Zhen, Huadong Pei, Suxia Han, Marcos Malumbres, Jianping Jin, Huan Chen, Yongxiang Zhao, Qing Zhu, Pumin Zhang

Nature Communications 8: Article number: 15751 ; DOI: 10.1038/ncomms15751 (2017); Published 06 12 2017; Updated 11 29 2017.

This Article contains an error in Fig. 7. In Fig. 7b, the label ‘siBRCRA1’ should have read ‘si53BP1’. The correct version of the figure appears below as Fig. 1.

## Figures and Tables

**Figure 1:**