# Supplementary material for: The anaphase promoting complex impacts repair choice by protecting ubiquitin signalling at DNA damage sites
Source: Nat Commun. 2017 Jun 12;8:15751. doi: 10.1038/ncomms15751 (PMC5472795; doi:10.1038/ncomms15751)
Supplement: Supplementary Information — Supplementary Figures [file ncomms15751-s1.pdf]

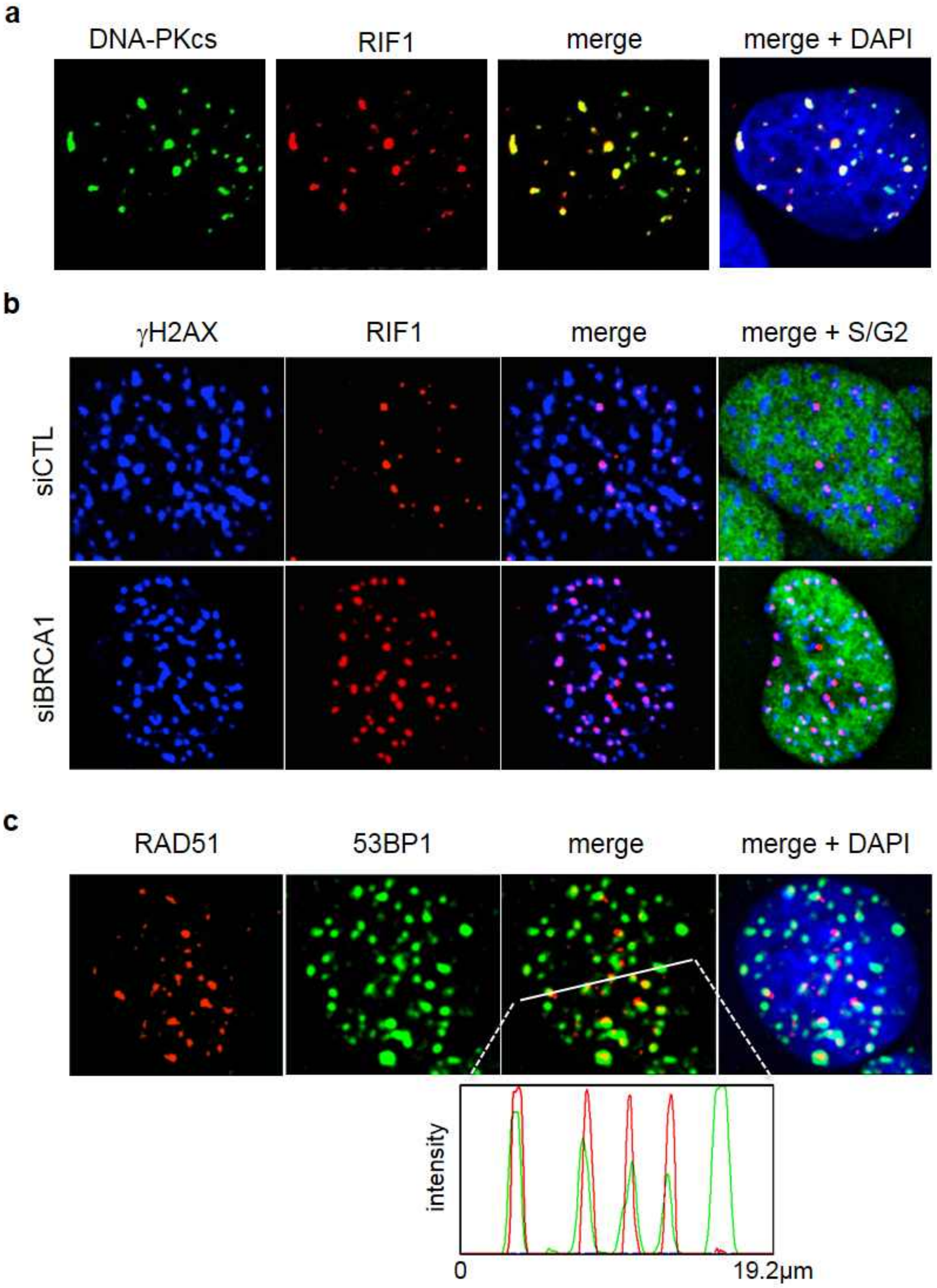

Supplementary Figure 1. IR-induced RIF1 and 53BP1 focus formation. (a) RIF1 colocalizes with DNA-PKsc, a factor required for NHEJ, in U2OS cells irradiated (2Gy) and recovered for 1 hr. (b) Three days after transfection of the indicated siRNAs, U2OS-Fucci cells were subjected to 2 Gy IR, and processed for immunostaining of γH2AX and RIF1 1 hr later. (c) U2OS cells were irradiated (2 Gy) and harvested for immunofluorescence staining 1 hr later.

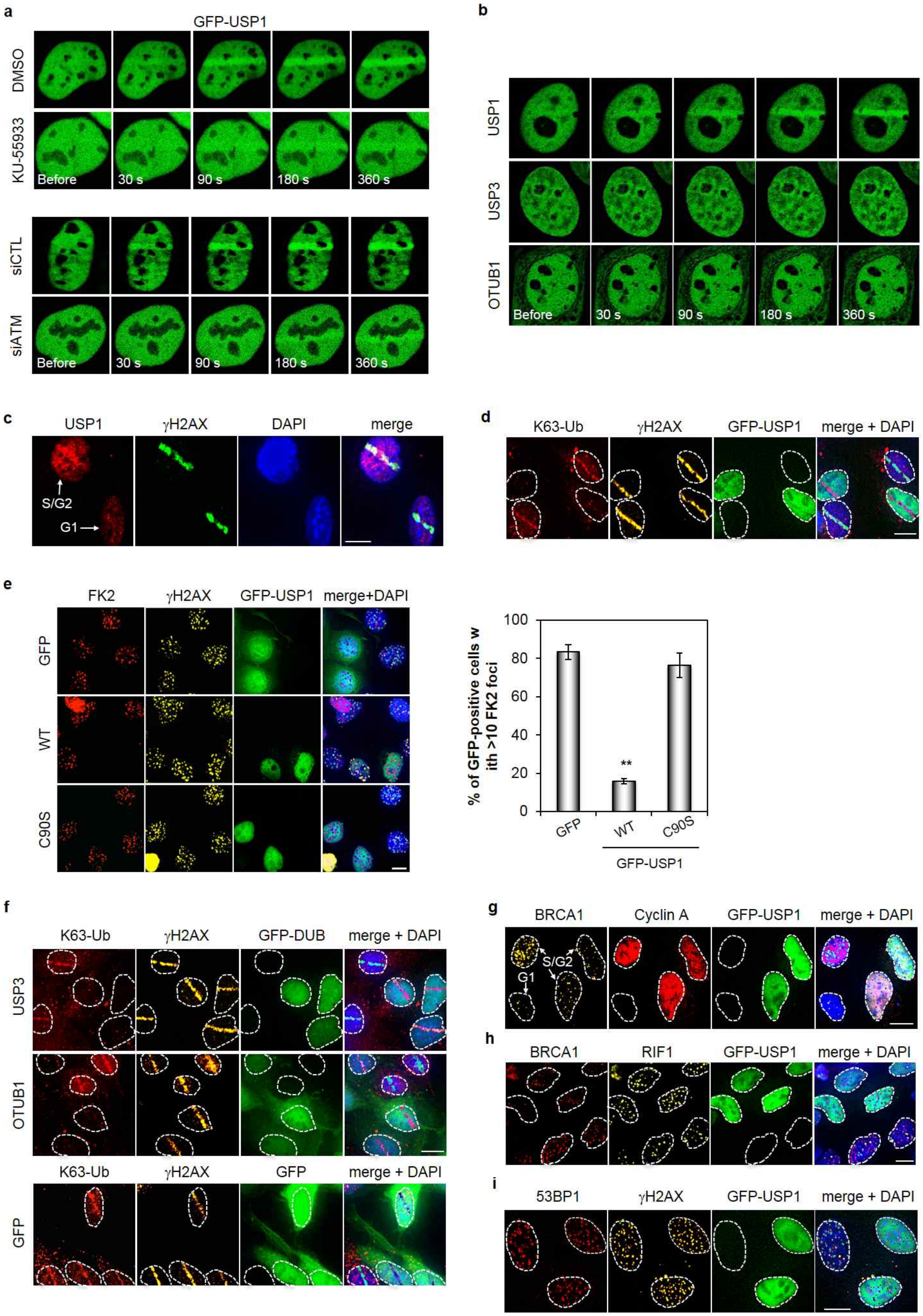
a
GFP-USP1
DMSO
KU-55933
Before
30 s
90 s
180 s
360 s
siCTL
siATM
b
USP1
USP3
OTUB1
c
USP1
γH2AX
DAPI
merge
S/G2
G1
d
K63-Ub
GFP-USP1
merge + DAPI
e
FK2
merge+DAPI
GFP
WT
C90S
% of GFP-positive cells with >10 FK2 foci
100
80
60
40
20
0
**
f
GFP-DUB
g
BRCA1
Cyclin A
h
RIF1
i
53BP1

j

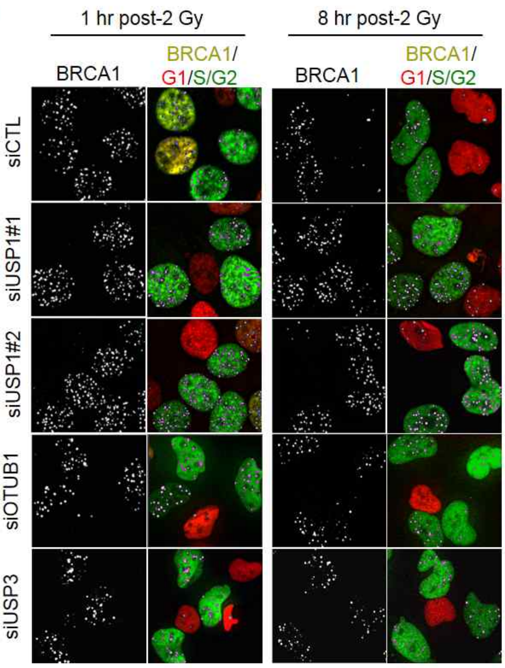
1 hr post-2 Gy
8 hr post-2 Gy
BRCA1
BRCA1/G1/S/G2
siCTL
siUSP1#1
siUSP1#2
siOTUB1
siUSP3

k

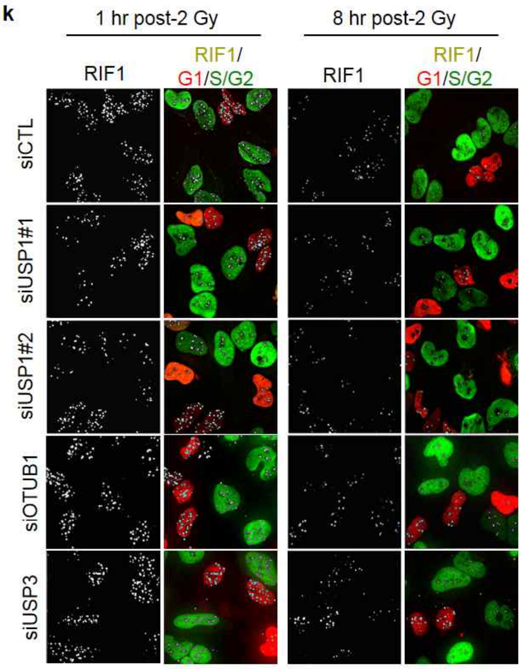
1 hr post-2 Gy
8 hr post-2 Gy
RIF1
RIF1/G1/S/G2
siCTL
siUSP1#1
siUSP1#2
siOTUB1
siUSP3

l

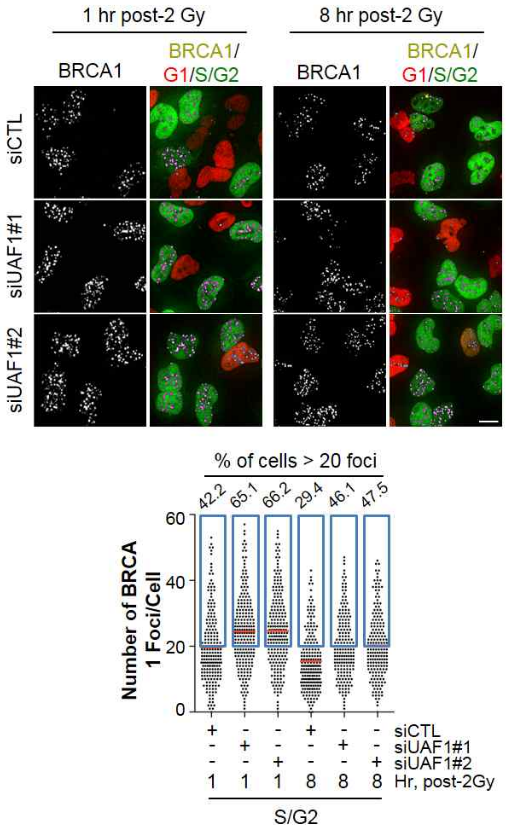
1 hr post-2 Gy
8 hr post-2 Gy
BRCA1
BRCA1/G1/S/G2
siCTL
siUAF1#1
siUAF1#2
% of cells > 20 foci
42.2
65.1
66.2
29.4
46.1
47.5
Number of BRCA 1 Foci/Cell
60
40
20
0
siCTL
siUAF1#1
siUAF1#2
Hr, post-2Gy
S/G2

m

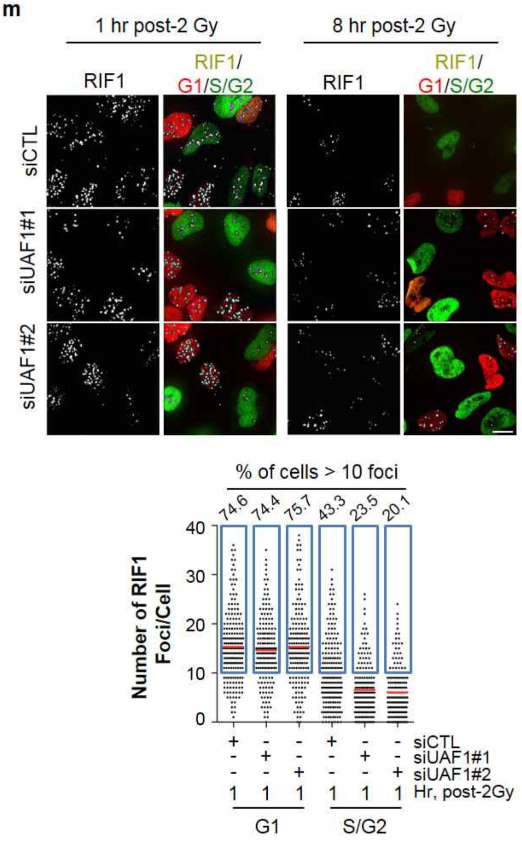
1 hr post-2 Gy
8 hr post-2 Gy
RIF1
RIF1/G1/S/G2
siCTL
siUAF1#1
siUAF1#2
% of cells > 10 foci
74.6
74.4
75.7
43.3
23.5
20.1
Number of RIF1 Foci/Cell
40
30
20
10
0
siCTL
siUAF1#1
siUAF1#2
Hr, post-2Gy
G1
S/G2

Supplementary Figure 2. USP1 prevents BRCA1 focus formation at DSBs in S/G2 cells by deubiquitinating histones. (a) The mobilization of GFP-USP1. U2OS cells were subjected to the indicated treatments, microirradiated, and imaged. (b) The visualization of USP1, USP3 and OTUB1 as GFP-tagged proteins following microirradiation in U2OS cells. (c) The accumulation of endogenous USP1 at microirradiated regions. U2OS cells were microirradiated, recovered for 10 min, and processed for immunostaining of USP1 and BRCA1. The cells with low USP1expression were in G1. (d) The effect of USP1 overexpression on K63-linked ubiquitin chains. U2OS cells transfected with GFP-USP1 were microirradiated, recovered for 30 min, and then processed for immunostaining of K63-linked ubiquitin and γH2AX. (e) The DUB activity of USP1 is essential in the removal of poly-Ub at damage sites. Left panel: co-staining of FK2 and γH2AX in U2OS cells transfected with USP1 expression constructs. Right panel: quantification of the data. (f) The effect of USP3 and OTUB1 overexpression on the formation of K63-linked ubiquitin. The experiment was performed similarly as in (d). (g-i) The effect of GFP-USP1 overexpression on BRCA1 (g), RIF1 (h) and 53BP1 (i) IRIF. U2OS cells transfected with GFP-USP1 were irradiated (2 Gy) and processed for immunostaining 1 hr after IR. (j and k) Immunostaining of BRCA1 (j) and RIF1 (k) in U2OS-Fucci cells transfected with the indicated siRNAs. 72 hours after the siRNA transfection, the cells were irradiated with 2 Gy, recovered for 1 hr, and harvested for immunostaining. (l and m) Representative images of BRCA1 IRIF (l) and RIF1 IRIF (m) in U2OS-Fucci cells transfected with the control and two different UAF1 siRNAs. Lower panels: quantification of BRCA1 (h, n>182 cells quantitated) and RIF1 (i, n>183 cells quantitated). Blue box designates cells with more than 20 (BRCA1 positive) and 10 (RIF1 positive) foci, respectively. The percentages of BRCA1 positive and RIF1 positive cells are indicated above each box. Error bars indicate SEM. ** indicates $p < 0.001$. Scale bars represent 10 μm.

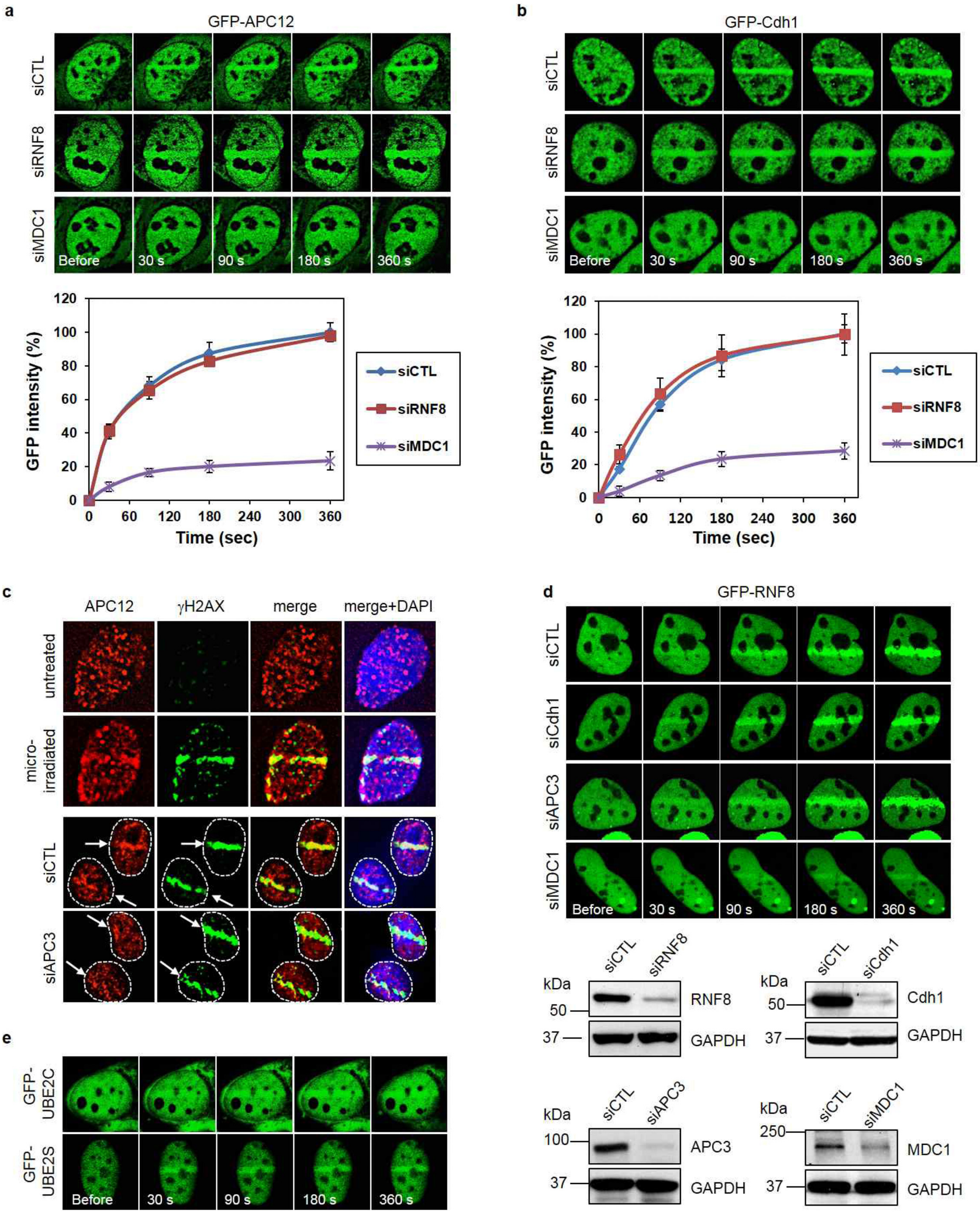
a
GFP-APC12
siCTL
siRNF8
siMDC1
Before
30 s
90 s
180 s
360 s
GFP intensity (%)
Time (sec)
0
60
120
180
240
300
360
20
40
60
80
100
120
siCTL
siRNF8
siMDC1
b
GFP-Cdh1
siCTL
siRNF8
siMDC1
Before
30 s
90 s
180 s
360 s
GFP intensity (%)
Time (sec)
siCTL
siRNF8
siMDC1
c
APC12
γH2AX
merge
merge+DAPI
untreated
micro-irradiated
siCTL
siAPC3
d
GFP-RNF8
siCTL
siCdh1
siAPC3
siMDC1
Before
30 s
90 s
180 s
360 s
siCTL
siRNF8
kDa
50
37
RNF8
GAPDH
siCTL
siCdh1
kDa
50
37
Cdh1
GAPDH
siCTL
siAPC3
kDa
100
37
APC3
GAPDH
siCTL
siMDC1
kDa
250
37
MDC1
GAPDH
e
GFP-UBE2C
GFP-UBE2S
Before
30 s
90 s
180 s
360 s

Supplementary Figure 3. The anaphase promoting complex localizes to sites of DNA damage in a MDC1-dependent, but RNF8-independent manner. (a and b) The mobilization of GFP-APC12 (a) and GFP-Cdh1 (b) in RNF8- and MDC1-depleted cells following microirradiation. Error bars indicate SEM. (c) The accumulation of endogenous APC12 at microirradiated regions. 72 hours after transfection of the indicated siRNAs, the cells were laser microirradiated and processed for APC12 and γH2AX immunostaining. (d) The mobilization of GFP-RNF8 in Cdh1-, APC3- and MDC1-depleted cells following microirradiation. The efficiency of siRNA-mediated target gene knockdown was shown by Western Blot analysis. (e) The mobilization of GFP-UBE2C and GFP-UBE2S to sites of DNA damage following microirradiation.

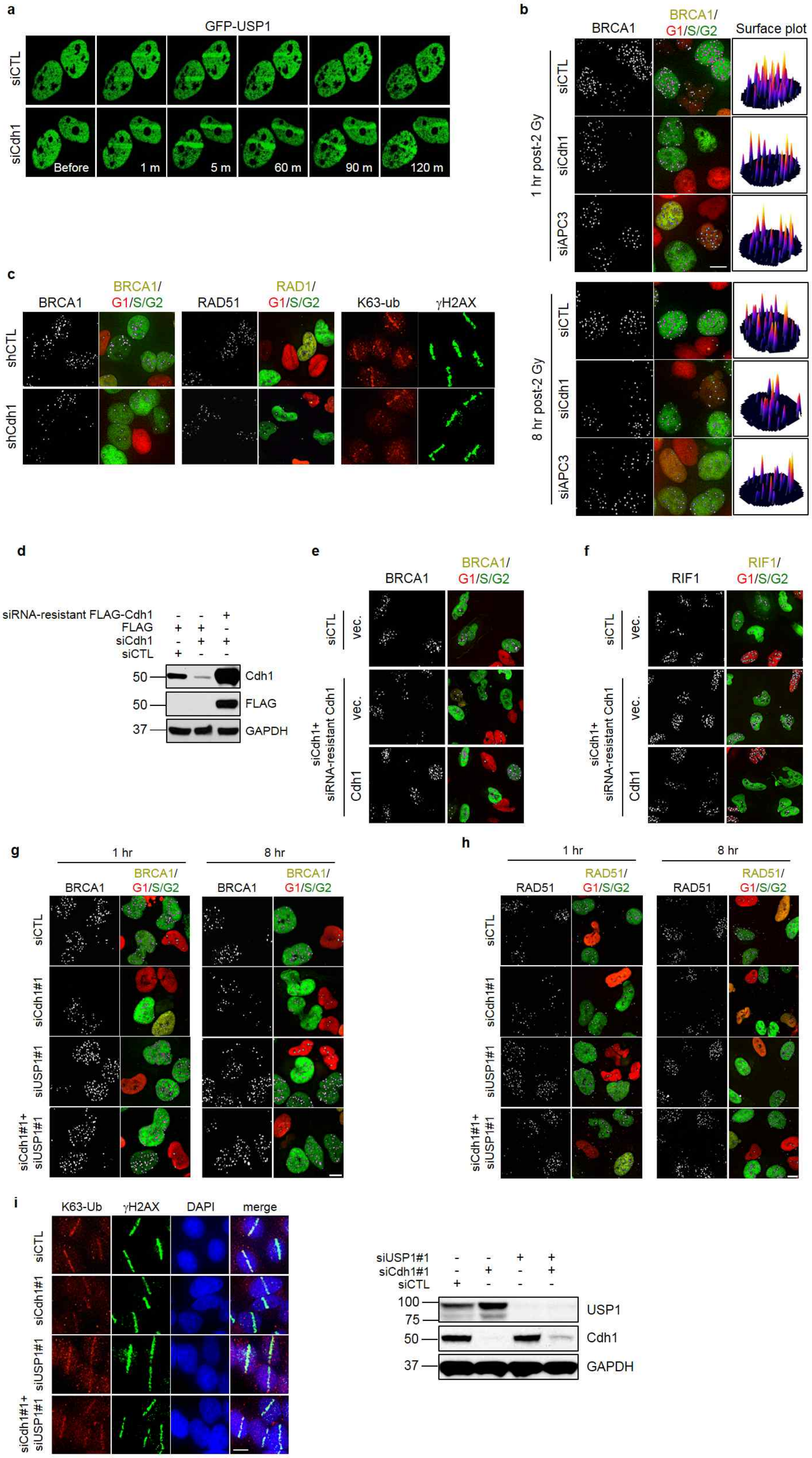
a
GFP-USP1
siCTL
siCdh1
Before
1 m
5 m
60 m
90 m
120 m
b
BRCA1
BRCA1/
G1/S/G2
Surface plot
1 hr post-2 Gy
8 hr post-2 Gy
siCTL
siCdh1
siAPC3
c
BRCA1
BRCA1/
G1/S/G2
RAD51
RAD1/
G1/S/G2
K63-ub
γH2AX
shCTL
shCdh1
d
siRNA-resistant FLAG-Cdh1
FLAG
siCdh1
siCTL
50
50
37
Cdh1
FLAG
GAPDH
e
BRCA1
BRCA1/
G1/S/G2
siCTL
vec.
siCdh1+
siRNA-resistant Cdh1
vec.
Cdh1
f
RIF1
RIF1/
G1/S/G2
siCTL
vec.
siCdh1+
siRNA-resistant Cdh1
vec.
Cdh1
g
1 hr
8 hr
BRCA1
BRCA1/
G1/S/G2
siCTL
siCdh1#1
siUSP1#1
siCdh1#1+
siUSP1#1
h
1 hr
8 hr
RAD51
RAD51/
G1/S/G2
siCTL
siCdh1#1
siUSP1#1
siCdh1#1+
siUSP1#1
i
K63-Ub
γH2AX
DAPI
merge
siCTL
siCdh1#1
siUSP1#1
siCdh1#1+
siUSP1#1
siUSP1#1
siCdh1#1
siCTL
100
75
50
37
USP1
Cdh1
GAPDH

Supplementary Figure 4. APC^{Cdh1} targets USP1 to protect poly-ubiquitin chains at DSB. (a) The mobilization of GFP-USP1 in Cdh1-depleted cells following microirradiation. U2OS cells were transfected with the indicated siRNAs and the next day with GFP-USP1. Microirradiation was performed after another 48 hour culturing. (b) Immunostaining of BRCA1 in U2OS-Fucci cells transfected with the indicated siRNAs. The cells were subjected to 2 Gy IR 3 days after the transfection, and harvested 1 and 8 hrs later for immunofluorescence analysis of BRCA1. (c) The inhibition of BRCA1 and RAD51 focus formation as well as K63-linked Ub chains in the cells depleted of Cdh1 by shRNA. U2OS and U2OS-Fucci cells were subjected to 2 Gy IR or to microirradiation 3 days after the transfection of the indicated shRNAs, and harvested for analyses 1 hr after the treatments. (d) The expression of siRNA-resistant FLAG-tagged Cdh1. 24 hrs after the transfection of the indicated siRNAs, U2OS cells were transfected with either FLAG only or FLAG-tagged Cdh1 expression vectors. Immunoblotting was performed after additional 48 hr culturing. (e and f) Representative images of BRCA1 (e) and RIF1 (f) IRIF in U2OS-Fucci cells transfected with the indicated siRNAs and expression vectors. The cells were irradiated (2 Gy) and processed for immunostaining 1 after IR. (g and h) Immunofluorescence analysis of BRCA1 (g) and RAD51 (h) in U2OS-Fucci cells transfected with the indicated siRNAs. The cells were irradiated (2 Gy) and processed for immunostaining 1 or 8 hrs after IR. (i) The regulation of K63 linkage Ub chain accumulation at DSBs by Cdh1. Immunofluorescence analysis of K63-linked Ub and γH2AX was performed following microirradiation in HeLa cells transfected with the indicated siRNAs. Right panel: the efficiency of siRNA-mediated target gene knockdown. Scale bars represent 10 μm.

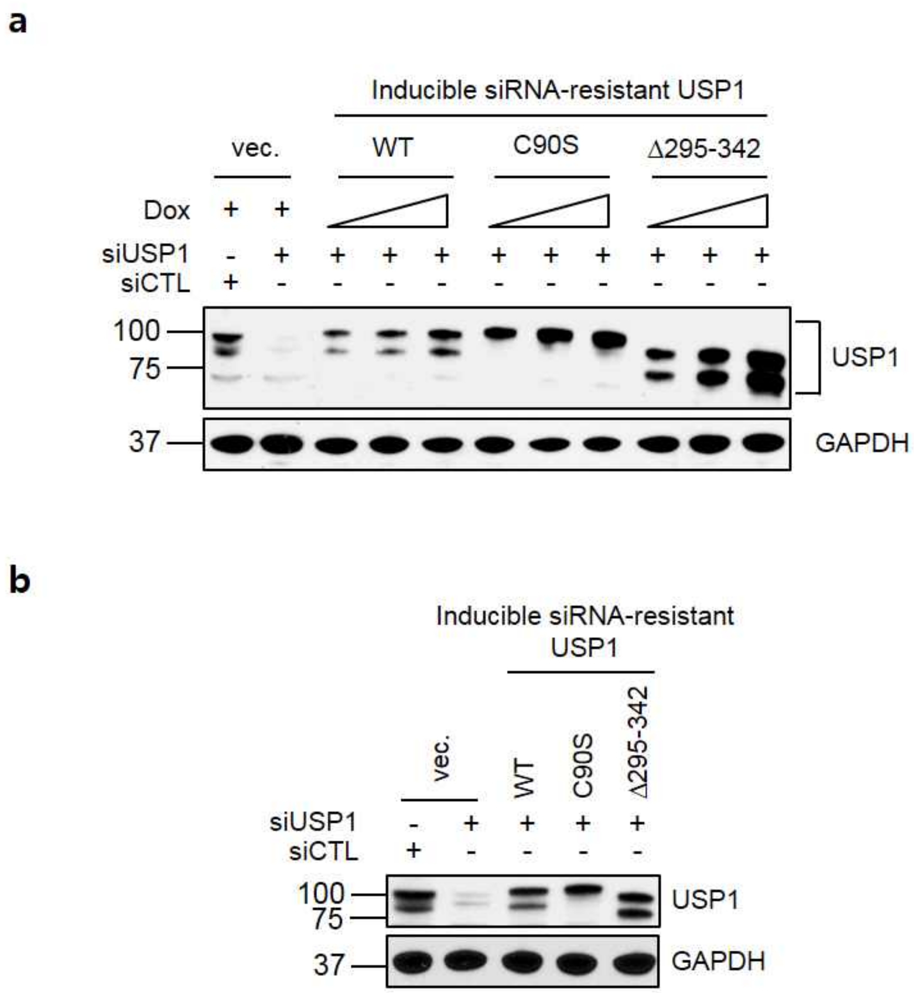

Supplementary Figure 5. Inducible expression of USP1. (a) Establishing conditions to induce siRNA-resistant USP1 expression at its endogenous level. The inducible expression was constructed in pInducer20. The constructs were packaged into lentiviruses and transduced into U2OS cells-Fucci cells. (b) Expression of siRNA-resistant USP1 wild type and mutants. Twenty four hours after the transfection of the indicated siRNAs, U2OS-Fucci cells stably expressing the indicated USP1 constructs were treated with doxycycline (0.01 mg/ml for WT, 0.05 μg/ml for C90S, and 0.1 μg/ml for Δ295-342) to induce the expression. Immunoblotting was performed after additional 48 hrs of culturing.

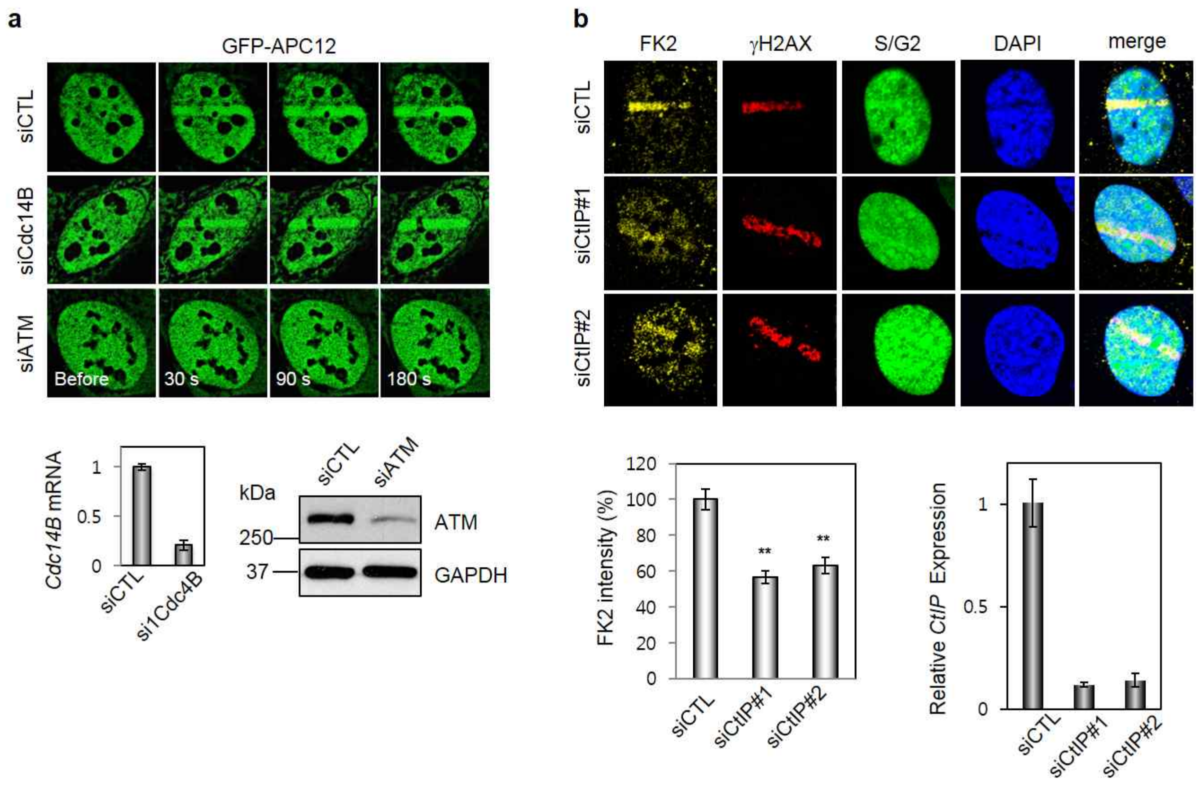

Supplementary Figure 6. The regulation of APC^{Cdh1} recruitment to the sites of DNA damage. (a) The mobilization of GFP-APC12 following microirradiation in U2OS cells depleted of Cdc14B or ATM was visualized. (b) Depletion of CtIP reduces poly-Ub chain formation at microirradiated regions. U2OS-Fucci cells were used. Data are represented as mean ± SEM of three independent experiments. Student's t-test was performed to determine the statistical significance. ** indicates $p < 0.001$.

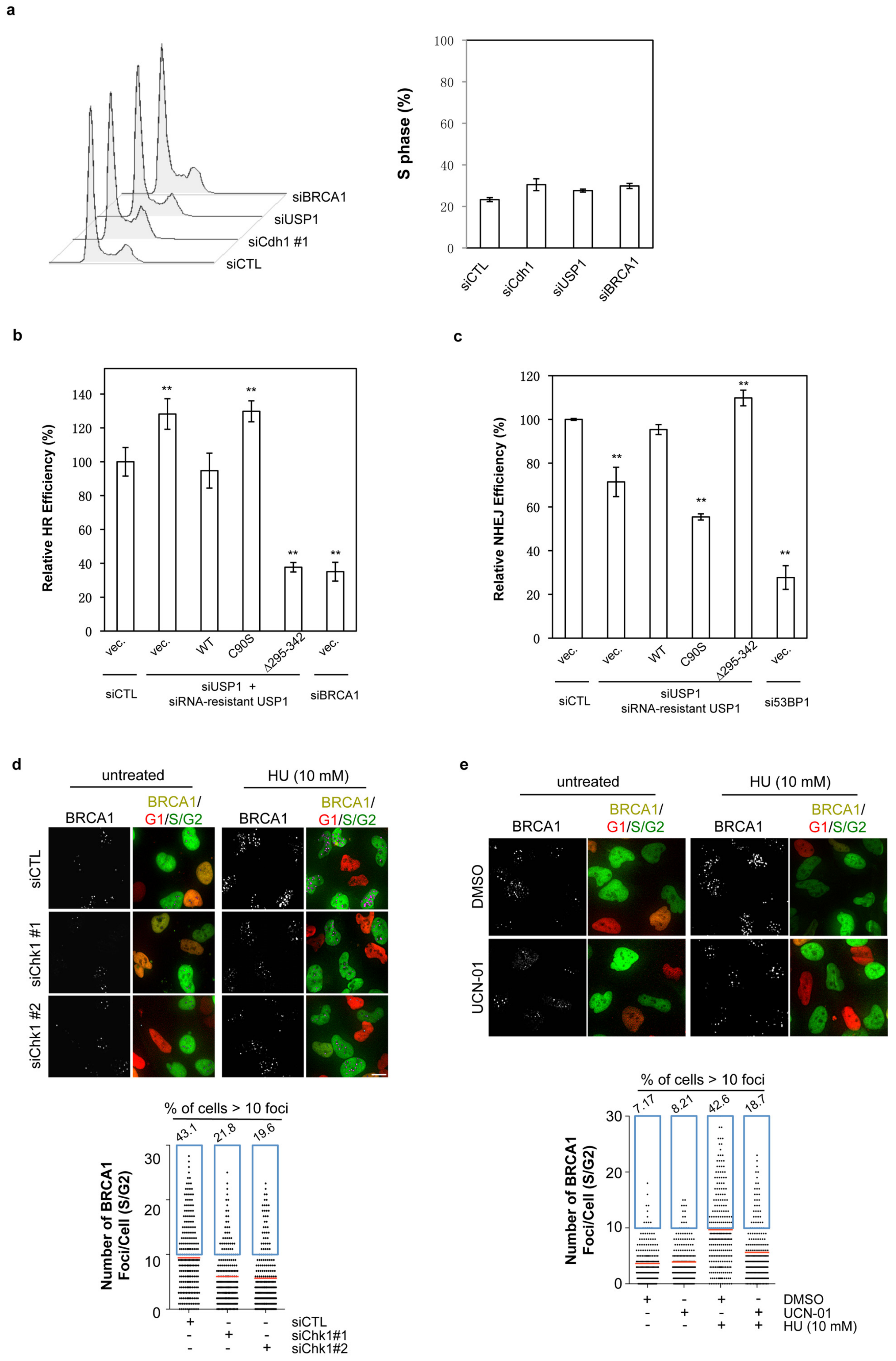
a
siBRCA1
siUSP1
siCdh1 #1
siCTL
S phase (%)
siCTL
siCdh1
siUSP1
siBRCA1
b
Relative HR Efficiency (%)
vec.
WT
C90S
Δ295-342
siCTL
siUSP1 +
siRNA-resistant USP1
siBRCA1
c
Relative NHEJ Efficiency (%)
siCTL
siUSP1
siRNA-resistant USP1
si53BP1
d
untreated
HU (10 mM)
BRCA1
BRCA1/
G1/S/G2
siCTL
siChk1 #1
siChk1 #2
% of cells > 10 foci
43.1
21.8
19.6
Number of BRCA1
Foci/Cell (S/G2)
siCTL
siChk1#1
siChk1#2
e
untreated
HU (10 mM)
DMSO
UCN-01
% of cells > 10 foci
7.17
8.21
42.6
18.7
DMSO
UCN-01
HU (10 mM)

Supplementary Figure 7. Chk1 is required for BRCA1 focus formation in response to replication stress. (a) FACS analyses of U2OS cells treated with siRNAs. (b, c) Quantification of HDR (b) and NHEJ (c) assays in USP1-depleted U2OS cells re-expressing wildtype, C90S, and D295-342 USP1. The HDR (the percentage of GFP+ cells among mCherry+ cells) and NHEJ (the percentage of tdTomato+ cells among GFP+ cells) efficiencies were normalized to that in control (siCTL) cells. (d) Quantitation of BRCA1 focus number per cell in U2OS-Fucci cells transfected with control and two different Chk1 siRNAs. 3 days after the siRNA transfection, the cells were treated with 10 mM of hydroxyurea for 4 hours and then harvested for immunostaining. Top panel: representative images of BRCA1 foci. Bottom panel: quantitation of BRCA1 focus number in the cells ($n>224$). (e) Quantitation of BRCA1 focus number per cell in U2OS-Fucci cells treated with DMSO or Chk1 inhibitor UCN-01 (300 nM). 24 hours after the treatment, the cells were treated with 10 mM of hydroxyurea for 4 hours and then harvested for immunostaining. Top panel: representative images of BRCA1 foci. Bottom panel: quantitation of BRCA1 focus number in the cells ($n>207$). Data are represented as mean ± SEM of three independent experiments. Student's t-test was performed to determine the statistical significance. ** indicates $p < 0.001$. Scale bars represent 10 μm.

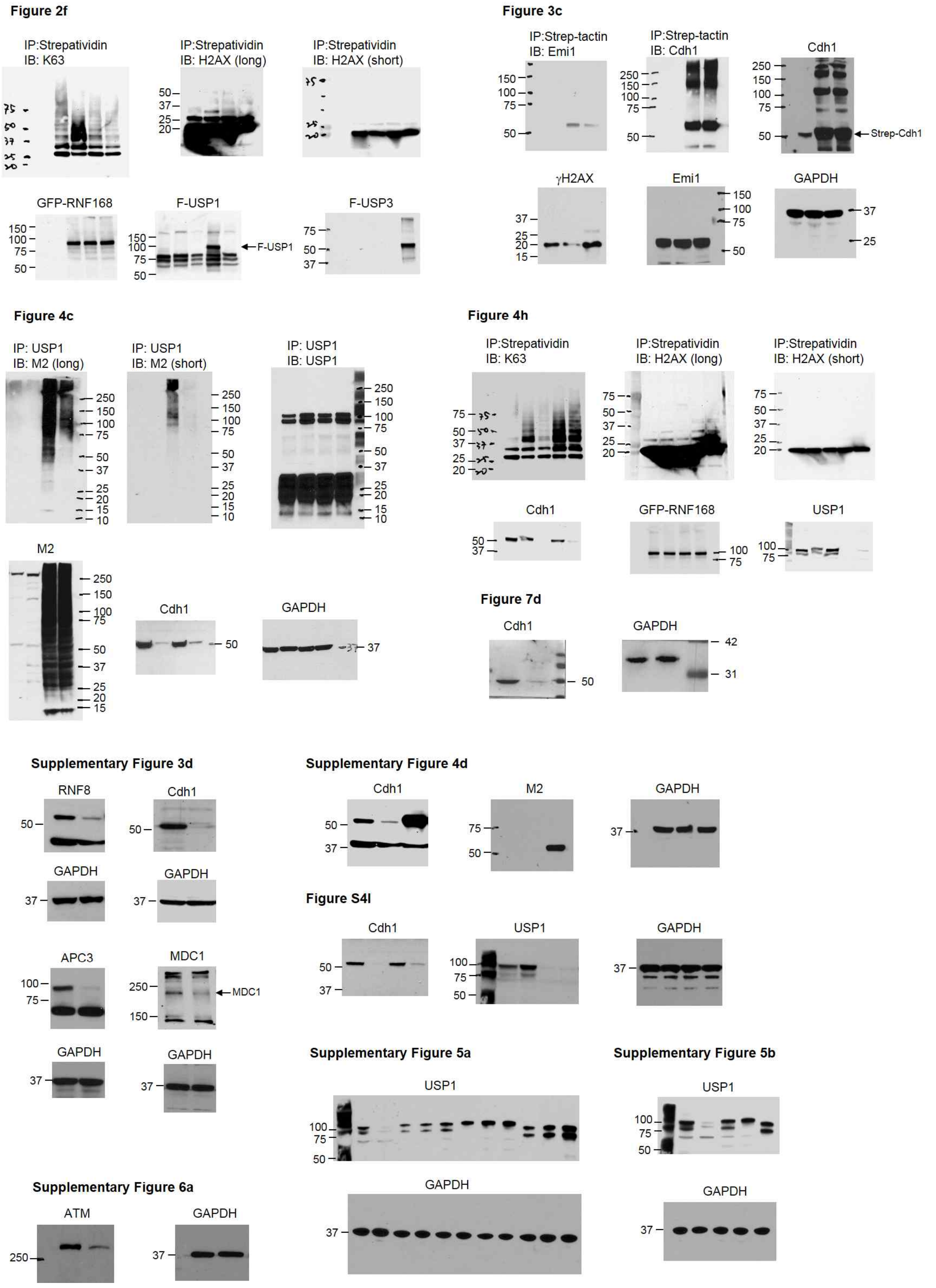

Supplementary Figure 8. Uncropped western blot images of Figures 2f, 3c, 4c,h, 7d and Supplementary Figures 3d, 4d, 5a,b, 6a.
